# Severe vivax malaria: a systematic review and meta-analysis of clinical studies since 1900

**DOI:** 10.1186/1475-2875-13-481

**Published:** 2014-12-08

**Authors:** Bilal Ahmad Rahimi, Ammarin Thakkinstian, Nicholas J White, Chukiat Sirivichayakul, Arjen M Dondorp, Watcharee Chokejindachai

**Affiliations:** Faculty of Tropical Medicine, Mahidol University, 420/6 Rajwithi Road, Bangkok, 10400 Thailand; Department of Pediatrics, Faculty of Medicine, Kandahar University, Kandahar, Afghanistan; Pacha Khan Academic Research Center, Kandahar University, Kandahar, Afghanistan; Section for Clinical Epidemiology and Biostatistics, Faculty of Medicine, Ramathibodi Hospital, Mahidol University, Bangkok, Thailand; Mahidol-Oxford Tropical Medicine Research Unit (MORU); Faculty of Tropical Medicine, Mahidol University, 3rd Floor, 60th Anniversary Chalermprakiat Building 420/6 Ratchawithi Road, Ratchathewi District, Bangkok, 10400 Thailand; Centre for Tropical Medicine and Global Health, Nuffield Department of Clinical Medicine, University of Oxford, Oxford, UK

**Keywords:** *Plasmodiumvivax*, Severe, Malaria, Complication, Prevalence, Systematic review, Meta-analysis

## Abstract

**Background:**

Malaria caused by *Plasmodium vivax* was long considered to have a low mortality, but recent reports from some geographical areas suggest that severe and complicated vivax malaria may be more common than previously thought.

**Methods:**

The primary objective of this systematic review and meta-analysis was to describe the reported clinical characteristics and the geographical variation in prevalence of reported severe vivax malaria and its change over time derived from English-language articles published since 1900. Medline and Scopus databases were searched for original papers on severe vivax malaria, using as inclusion criteria modified 2010 WHO criteria for the diagnosis of severe falciparum malaria. Articles before 1949 were identified through reference lists in journals, textbooks, and personal collections of colleagues.

**Results:**

A total of 77 studies with reported severe vivax malaria and 63 studies with no reported severe vivax malaria (totaling 46,411 and 6,753 vivax malaria patients, respectively) were included. The 77 studies with reported severe vivax malaria were mainly from India (n = 33), USA (n = 8), Indonesia (n = 6), and Pakistan (n = 6). Vivax endemic countries not reporting severe vivax malaria beyond individual case reports included: the Greater Mekong Sub-region, China, North Korea, Bangladesh, Afghanistan, Middle East (except Qatar), the horn of Africa, and Madagascar. Only 17/77 reports were from before 2000. Vivax mono-infection was confirmed by PCR in 14 studies and co-morbidities were ruled out in 23 studies. Among the 77 studies reporting severe vivax malaria, severe thrombocytopenia (<50,000/mm^3^) was the most common “severe” manifestation (888/45,775 with pooled prevalence of 8.6%). The case fatality was 0.3% (353/46,411). Severity syndromes varied widely between different geographical areas, with severe anaemia being most prominent in areas of high transmission and chloroquine resistance.

**Conclusion:**

*Plasmodium vivax* can cause severe and even fatal disease, but there is a recent increase in reports over the past 15 years with larger series restricted to a limited number of geographical areas. The biological basis of these variations is currently not known. More detailed epidemiological studies are needed which dissociate causation from association to refine the definition and estimate the prevalence of severe vivax malaria.

**Electronic supplementary material:**

The online version of this article (doi:10.1186/1475-2875-13-481) contains supplementary material, which is available to authorized users.

## Background

Of the five *Plasmodium* species causing disease in humans, *Plasmodium falciparum* is the main cause of severe and fatal disease. Traditionally *Plasmodium vivax* has been considered relatively benign, except for occasional severe manifestations notably severe anaemia and the acute respiratory distress syndrome [1]. Although death in *P. vivax* infections has been recognized for over a century, the last decade has seen a remarkable increase in case reports, series and studies describing severe and fatal vivax malaria. An estimated 2.8 billion people globally live in one of the 95 countries endemic for *P. vivax*[2–5]. In general, in *P. vivax* endemic areas transmission is low and seasonal (Central, West, South, and South-East Asia), the Americas, and the horn of Africa (and Madagascar); whereas the rest of Africa is relatively spared because of the absence of the Duffy blood group which mediates parasite invasion [5]. In contrast transmission is substantially higher on the island of New Guinea. India contributes nearly half (46%) , China 19%, while Indonesia and Pakistan together contribute 12% of the global population at risk [5]. India has the majority of clinical cases.

Occasional fatalities in vivax malaria have been reported since the species was first recognized, particularly in already debilitated patients, and significant mortality has been attributed to vivax malaria in the first and second world wars although details of speciation are often scanty. *Plasmodium vivax* was associated with a 7.7% mortality in the malariatherapy of neurosyphilis, but this was attributed in part to the debilitated condition of the patients and mortality in *Plasmodium malariae* infections was even higher [6]. Early publications reporting severe vivax malaria include case reports [7–9] and small case series [10, 11], but in recent years, larger studies conducted in India, Indonesian Papua, and Papua New Guinea suggest a stronger association between *P. vivax* infection, severe disease and death than recognized previously [12–16]. A systematic review and meta-analysis was conducted from all identifiable English-language articles published since 1900 reporting human cases of severe *P. vivax* infection. The primary objective was to describe the geographical variation in prevalence of severe vivax malaria and the different presenting syndromes and characteristics, as well as to analyse the changes in reporting over time.

## Methods

### Search engines, terms, and strategies

Eligible studies on severe vivax malaria since 1949 were identified in Medline (via PubMed) and Scopus (via Scopus). The final search and updates in both databases were carried out on January 28, 2014. Articles before 1949 (Pre-PubMed era) were mainly searched through other sources including reference lists of published journal articles and (old) textbooks and through gifts of personal collections of colleagues. Details on variables, search terms, and search strategies are explained in Additional file 1.

### Inclusion/exclusion criteria

All literature in English describing clinical studies in human vivax malaria was considered, recognizing that there is also a substantial literature in other languages. Malaria was diagnosed by peripheral blood smear (PBS) and in recent years by rapid diagnostic test (RDT) or polymerase chain reaction (PCR). Since severity criteria for severe vivax malaria have not been specified yet, modified criteria defined in the 2010 WHO supplement on severe falciparum malaria were used as the outcome of interest used for inclusion, with the addition of thrombocytopenia as this has been used as a severity criterion in several publications (<50,000/mm^3^) [17] [see Additional file 2]. It should be noted that thrombocytopenia is not a severity criterion for falciparum malaria, and has never been validated as an independent severity measure in vivax malaria. Only articles reporting *P. vivax* mono-infection were selected. Studies included those where the denominators were both inpatients and outpatients with vivax malaria and studies reporting only inpatients with vivax malaria patients. Exclusion criteria included duplicate reporting, mixed infections (*P. vivax* with any other *Plasmodium* species), and insufficient data for extraction. All types of study designs with primary data (having patients of either gender or any age) were eligible, but case reports and case series were not included in pooled calculations of prevalence because the denominator was uncertain. The complete selected articles were analysed and data were coded into a data extraction form (DEF).

### Bias assessment and quality assurance

Eligibility for inclusion of every article was reviewed by independent reviewers (Bilal Ahmad Rahimi, Wali Mohammad Wyar, and Arjen M. Dondorp). Disagreements between reviewers were resolved by discussion, and extracted data were validated by an independent person (Watcharee Chokejindachai).

### Statistical analysis

For the meta-analysis, pooled prevalence was calculated as


where  = Pooled prevalence of the severity signs among vivax malaria patients;

p_i_ = Prevalence of the severity sign in each study;

w_i_ =1/var (p_i_), which was the weight of each study.

The pooled prevalence and 95% CI of each severity sign was calculated only if it was reported in at least three reports using the following command in STATA


where n = Total number of patients with a specific severity sign due to vivax malaria; freq = Total number of patients with vivax malaria in the study.

A subgroup analysis was performed in order to compare prevalence of severity signs before and after 2000 and between different WHO regions (Africa [AFRO], Americas [AMRO], Eastern-Mediterranean [EMRO], Europe [EURO], South-East Asia [SEARO], and Western Pacific [WPRO]) and countries, using Chi^2^ test. Software STATA version 12 (Stata Corp LP, Texas USA) was used for statistical analysis.

## Results

**Figure 1:**
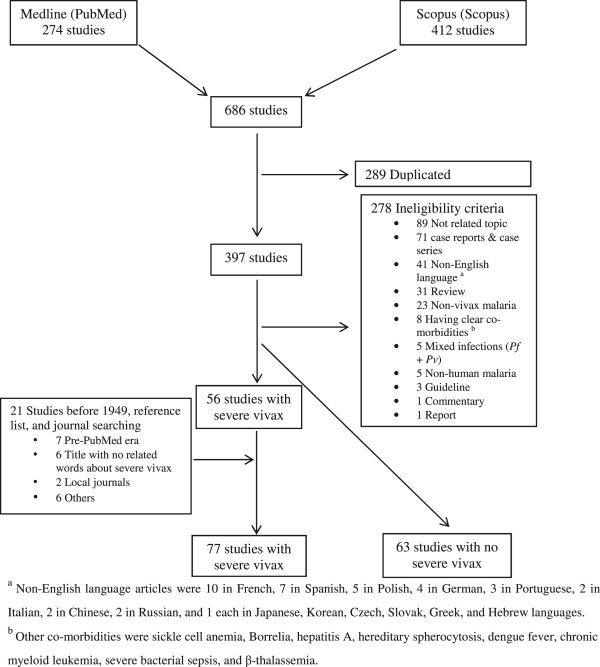
**Flow of study selection.**

Initially 274 and 412 studies were identified in the Medline and Scopus databases, respectively. Of these, 289 were duplicates and 278 were ineligible for other reasons, including: only case reports and case series (n = 71), non-English language (n = 41), and disease caused by obvious co-morbidities (n = 8) (Figure 1). Twenty-one additional studies (including studies from before 1949) were included through search of reference lists or from reference files provided by colleagues, providing a total of 77 studies with severe vivax malaria patients and 63 studies with no severe vivax malaria patients, comprising a total of 46,411 and 6,753 vivax malaria patients, respectively.

### Characteristics of studies reporting severe vivax malaria

Characteristics of the 77 studies with severe vivax malaria patients are explained in Additional file 3. Among them, 36 were retrospective hospital-based studies (RHBS) and 41 were prospective hospital-based studies (PHBS). Regarding their geographical origin, 42 (54.5%), 17 (22.1%), 10 (13%), and 8 (10.4%) were from SEARO, AMRO, EMRO, and WPRO, respectively. Specified by country, 33 (42.9%), 8 (10.39%), 6 (7.8%), and 6 (7.8%) studies were reported from India, USA, Pakistan, and Indonesia, respectively.

### Pooled prevalence of severity signs from 140 studies reporting on both severe and uncomplicated vivax malaria

Overall 77 studies reported patients with severe vivax malaria patients while 63 studies did not report any patients as having severe vivax malaria.

**Figure 2:**
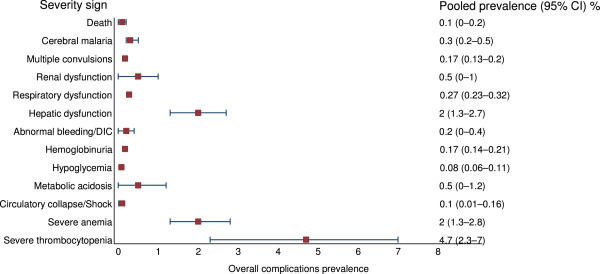
**Forest plot of pooled prevalences of the severity signs in** ***P. vivax*** **malaria patients in studies with and without reported severe vivax malaria patients (140 studies).**

The pooled prevalence of the various severity signs in studies with and without reported severe vivax malaria patients are shown in Table 1 and Figure 2. Among these studies, the pooled prevalence of the five most commonly reported severity signs were: severe thrombocytopenia 4.7% (95% CI: 2.3–7%), severe anaemia 2% (95% CI: 1.3–2.8%), hepatic dysfunction 2% (95% CI: 1.3–2.7%), metabolic acidosis 0.5% (95% CI: 0–1.2%), and renal dysfunction 0.5% (95% CI: 0–1%). Pooled mortality was 0.1% (95% CI: 0–0.2%).

**Table 1:** **Pooled prevalence of the various severity signs in studies with and without reported severe vivax malaria patients (140 studies)**

Complication	Total vivax	Total patients with severity sign	Pooled prevalence, %	95% CI, %
Death	53164	353	0.1	0–0.2
Cerebral malaria	52598	532	0.3	0.2–0.5
Multiple convulsions	53139	88	0.17	0.13–0.2
Renal dysfunction	53094	244	0.5	0–1
Respiratory dysfunction	53164	144	0.27	0.23–0.32
Hepatic dysfunction	53289	727	2	1.3–2.7
Abnormal bleeding/DIC	53099	206	0.2	0–0.4
Haemoglobinuria	53164	93	0.17	0.14–0.21
Hypoglycaemia	53134	44	0.08	0.06–0.11
Metabolic acidosis	53094	138	0.5	0–1.2
Circulatory collapse/Shock	53164	67	0.1	0.01–0.16
Severe anaemia	53164	2276	2	1.3–2.8
Severe thrombocytopenia	52528	888	4.7	2.3–7

### Pooled prevalence of severity signs from 77 studies reporting on severe vivax malaria only

**Figure 3:**
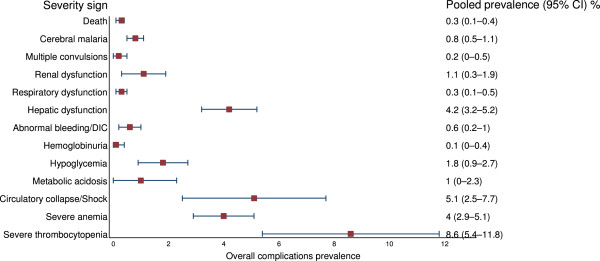
**Forest plot of pooled prevalences of the severity signs in** ***P. vivax*** **malaria patients in studies with reported severe vivax malaria patients (77 studies).**

Among 77 studies with reported severe vivax malaria patients, 43 reported hepatic dysfunction, 42 cerebral malaria, 37 deaths, 34 severe anaemia, 27 severe thrombocytopenia, 27 respiratory dysfunction, 24 abnormal bleeding/DIC, 20 renal dysfunction, 14 hypoglycaemia, 11 generalized seizures, 11 circulatory collapse/shock, 10 haemoglobinuria, and eight studies reported metabolic acidosis. The pooled prevalence of the various severity signs in studies with reported severe vivax malaria patients are shown in Table 2 and Figure 3. Among these studies, the pooled prevalence of the five most commonly reported severity signs were: severe thrombocytopenia 8.6% (95% CI: 5.4–11.8%), shock 5.1% (95% CI: 2.5–7.7%), hepatic dysfunction 4.2% (95% CI: 3.2–5.2%), severe anaemia 4% (95% CI: 2.9–5.1%), and hypoglycaemia 1.8% (95% CI: 0.9–2.7%). Pooled mortality was 0.3% (95% CI: 0.1–0.4%). Detailed analysis of pooled prevalence of severity signs among reported severe vivax malaria patients are shown in Additional file 4.

**Table 2:** **Pooled prevalence of severity signs among reported severe vivax malaria patients (77 studies)**

Complication	Total vivax	Total patients with severity sign	Pooled prevalence, %	95% CI, %
Death	46411	353	0.3	0.1–0.4
Cerebral malaria	45845	532	0.8	0.5–1.1
Multiple convulsions	46386	88	0.2	0–0.5
Renal dysfunction	46341	244	1.1	0.3–1.9
Respiratory dysfunction	46411	144	0.3	0.1–0.5
Hepatic dysfunction	46536	727	4.2	3.2–5.2
Abnormal bleeding/DIC	46346	206	0.6	0.2–1
Haemoglobinuria	46411	93	0.1	0–0.4
Hypoglycaemia	46381	44	1.8	0.9–2.7
Metabolic acidosis	46341	138	1	0–2.3
Circulatory collapse/Shock	46411	67	5.1	2.5–7.7
Severe anaemia	46411	2276	4	2.9–5.1
Severe thrombocytopenia	45775	888	8.6	5.4–11.8

### Comparisons with falciparum malaria

In Indonesian Papua, coma associated with PCR-confirmed *P. vivax* mono-infection (and without overt co-morbidities) occurred 23 times less frequently than that seen with falciparum malaria and was estimated as occurring in one in 29,500 infections [18]. In Thailand, the risk of hospitalization with impaired consciousness with microscopy-diagnosed *P. vivax* (not PCR-confirmed) was one in 858 infections, with the risk being 15.2 fold less than that with *P. falciparum*[19]. Mortality was 0.22% in children hospitalized in Eastern Thailand with vivax malaria (concomitant falciparum malaria excluded by microscopy only) [20].

### Pooled prevalence of severity signs in vivax malaria from 62 studies reporting on both inpatients and outpatients

Among 77 studies with severe vivax malaria patients, 62 studies reported severity signs in both inpatients and outpatients of vivax malaria. In these studies, 31 reported cerebral malaria, six generalized seizures, 14 renal dysfunction, 19 respiratory dysfunction, 31 hepatic dysfunction, 17 abnormal bleeding/DIC, seven haemoglobinuria, eight hypoglycaemia, four metabolic acidosis, six circulatory collapse/shock, 23 severe anaemia, 21 severe thrombocytopenia, and 28 studies reported fatal cases [see Additional files 5, 6, 7, 8, 9, 10, 11, 12, 13, 14, 15, 16 and 17].

**Figure 4:**
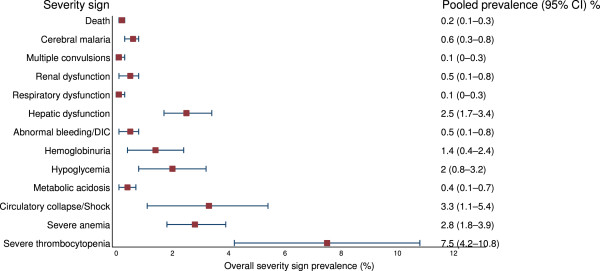
**Forest plot of pooled prevalences of the severity signs in** ***P. vivax*** **malaria patients in studies that reported both inpatients and outpatients of** ***P. vivax*** **malaria (62 studies).**

The pooled prevalence of the various severity signs in studies that reported both inpatients and outpatients of vivax malaria are shown in Additional file 18 and Figure 4. The pooled prevalence of the five most commonly reported severity signs were: severe thrombocytopenia 7.5% (95% CI: 4.2–10.8%), shock 3.3% (95% CI: 1.1–5.4%), severe anaemia 2.8% (95% CI: 1.8–3.9), hepatic dysfunction 2.5% (95% CI: 1.7–3.4%), and hypoglycaemia 2% (95% CI: 0.8–3.2%). Pooled mortality was 0.2% (95% CI: 0.1–0.3%).

### Pooled prevalence of severity signs in vivax malaria from 15 studies reporting on inpatients only

Among 77 studies with severe vivax malaria patients, 15 studies described severity signs only in inpatients vivax malaria. In these studies, 11 reported cerebral malaria, 5 repeated generalized seizures, 6 renal dysfunction, 8 respiratory dysfunction, 12 hepatic dysfunction, 7 abnormal bleeding/DIC, 3 haemoglobinuria, 6 hypoglycaemia, 4 metabolic acidosis, 5 circulatory collapse/shock, 10 severe anaemia, 6 severe thrombocytopenia, and 9 studies reported deaths [see Additional files 19, 20, 21, 22, 23, 24, 25, 26, 27, 28, 29, 30 and 31].

**Figure 5:**
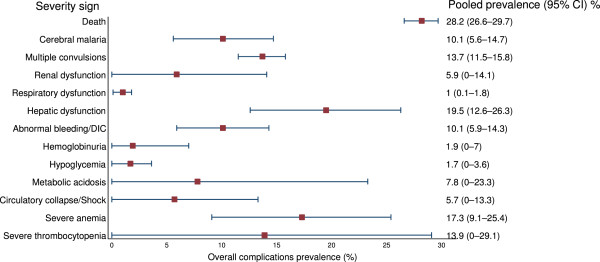
**Forest plot of pooled prevalence of the severity signs in** ***P. vivax*** **malaria patients in studies that reported only inpatients of** ***P. vivax*** **malaria (15 studies).**

The pooled prevalence of severity signs among reported inpatients with vivax malaria are shown in Additional file 32 and Figure 5. The five most common severity manifestations were death 28.2% (95% CI: 26.6–29.7%), hepatic dysfunction 19.5% (95% CI: 12.6–26.3%), severe anaemia 17.3% (95% CI: 9.1–25.4%), and severe thrombocytopenia 13.9% (95% CI: 0–29.1%).

### Comparison of severity signs between vivax and falciparum malaria

**Figure 6:**
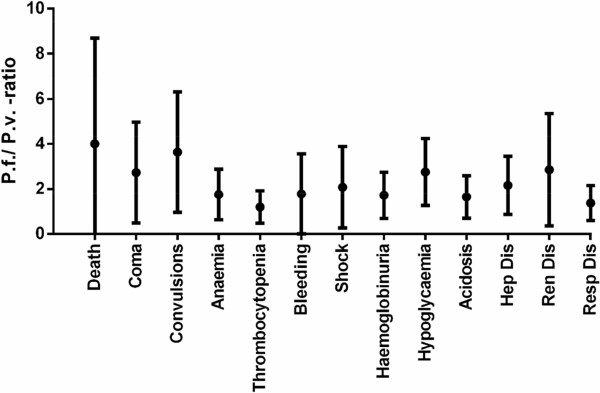
***Pf*** **:** ***Pv*** **ratio of severity signs in** ***P. vivax*** **and** ***P. falciparum*** **malaria patients.**

Figure 6 shows the *Pf:Pv* ratio for all signs of malaria severity. Highest mean *Pf:Pv* ratio was present for fatalities (4.0), followed by repeated generalized seizures (3.64), renal dysfunction (2.85), hypoglycaemia (2.75), and cerebral malaria (2.73). Lowest mean *Pf:Pv* ratio was for severe thrombocytopenia (1.19) and respiratory dysfunction (1.37).

## Discussion

There is substantial variation in the reported geographic distribution and the incidence of severe manifestations of vivax malaria. This analysis also shows a large increase since the year 2000 in the number of studies and numbers of patients reported with severe *P. vivax* infections, (which in most studies was confirmed as mono-infection by PCR). Of these 33 (42.9%) studies came from India. In contrast, a large number of endemic countries do not report severe vivax malaria, including the Greater Mekong Sub-region, North-east China, North-Korea, Bangladesh, Afghanistan, Middle East (except Qatar), Somalia, and Madagascar. There are two general presentations reported; severe anaemia in young children from the island of New Guinea where transmission is high, and vital organ dysfunction usually in adults from low transmission settings. A large variation in organ involvement was observed in the latter group. Vivax malaria was reported as an important cause of hospital admission in endemic areas, but a major problem in determining the true incidence and prevalence of severe vivax malaria is that severe disease has been considered relatively unusual in vivax malaria, and so its absence from a clinical series has not been considered noteworthy. For example in over 1,000 patients with severe malaria admitted to a centre in Ho Chi Minh city, Vietnam, specializing in its management there was one case of severe vivax (cerebral) malaria and no cases of vivax malaria associated acute renal failure (TT Hien & NP Phu: personal communication), but this paucity of severe disease was not considered noteworthy and so not reported. This contrasts with a prevalence of severe disease amongst admitted cases of over 30% in some series. Thus estimating incidence and prevalence from series which do report severe vivax malaria provides overestimates. Furthermore the most prevalent severity sign reported was thrombocytopenia <50,000/μL (26.1%), which is not regarded as a severity sign in falciparum malaria, and has not been validated as an independent severity measure in vivax malaria. The least prevalent manifestation was haemoglobinuria (0.1%). In studies with only inpatients of vivax malaria, thrombocytopenia (35.9%) was the most commonly reported severity sign, while least prevalent severity sign was respiratory dysfunction (1%). Mortality in studies with only inpatients of vivax malaria was 28.2%, compared to 0.2% in groups with both inpatients and outpatients of vivax malaria.

### How can the sharp increase in reported cases of severe vivax malaria, the geographical heterogeneity, and the different severe manifestations be explained?

It seems unlikely that underreporting can explain the scarcity of cases in the last century, although there are only few data to support this. A study in American and Allied soldiers in India reported in 1944, described cerebral malaria in 5/1,375 cases (0.4%) with *P. vivax*, compared to 93/2664 (3.5%) with *P. falciparum* or mixed infection [21]. An Indian study from 1982 reported severe malaria in 4/178 (2.2%) patients with *P. vivax* compared to 64/382 (17%) with *P. falciparum* or mixed infection [22]. A more recent Indian study showed a substantially higher proportion of patients (50/338 (15%)) having severe vivax malaria [16]. One contributor to this discrepancy is the inclusion of thrombocytopenia as a severity criterion in recent series.

The geographical heterogeneity is more substantiated by data; a study from the Thai-Myanmar border from 1997 describes a very low incidence of patients with severe *P. vivax* malaria (3 out of 2573 patients) [19]. A case of *P. vivax*-related pulmonary oedema has been reported from this area [23].

### Can the increase in chloroquine resistance account for the observed geographical differences?

Chloroquine resistance has been well documented on the Island of New Guinea (Papua New Guinea [24] and Indonesian Papua [25, 26] and more recently in Amazonas [27]. All these regions report severe vivax malaria. On the other hand, chloroquine resistance has not been clearly described in other areas also reporting severe cases, including India [28–31] and Pakistan [32, 33], although study methodology was not optimal. Chloroquine resistance in settings where chloroquine is still widely used will extend the duration of illness considerably, and recurrent infection is expected to result in an increase in patients presenting with severe anaemia. Indeed, severe anaemia is by far the most common presentation in PNG and Indonesia (a high transmission setting where children are affected predominantly), but less so in Amazonia (a low transmission setting where adults are affected predominantly). Compounding factors to severe anaemia include falciparum malaria, intestinal helminths and nutritional deficiencies [34, 35]. A retrospective study in Indonesian Papua reported similar adjusted odd ratios of death associated with severe anaemia (Hb <5 g/dl) of 4.43 and 5.93 for *P. vivax* and *P. falciparum*, respectively. In the same population patients with both falciparum and vivax malaria and thrombocytopenia <50,000/mm^3^ had an increased mortality, although as not all the other clinical and laboratory measures used to assess disease severity were recorded it is unclear whether thrombocytopenia was an independent risk factor or not [36]. In a study by Limaye and colleagues in Mumbai, India, the most common severe manifestation causing death was ARDS (six out of six patients) [16].

### Can the heterogeneity be explained by differences in background *P. vivax*parasitaemia, resulting in misdiagnosis of severe febrile illnesses with coincidental *P. vivax*parasitaemia, analogous to the overdiagnosis of falciparum malaria as a cause of illness in parasitaemic African children?

There are few data available, but in Bikaner (India) where many of the Indian reports on severe malaria originate, the population prevalence of vivax malaria is only around 1% (Maude RE, personal communication). In contrast, the population prevalence is higher in endemic regions Myanmar [37, 38], which does not report severe vivax. In this review, 23/77 (29.9%) studies attempted to rule out possible co-morbidities as confounders. One possibility is that other severe febrile illnesses could reactivate hepatic hypnozoites resulting in relapse. High vivax relapse rates, up to over 40%, have been observed following *P. falciparum* infection [39–41]. Of the reported studies, only 11 (18.6%) studies used PCR for confirmation of *P. vivax* mono-infection, but even this would not rule out recently cleared falciparum infection. Typhoid fever, relapsing fever, trench fever, epidemic typhus, and brucellosis have been incriminated as activating *P. vivax* hypnozoites [42]. In many of the published studies there were only limited efforts to rule out other diseases, including falciparum malaria, bacterial diseases, and viral diseases [13, 43, 44]. Regions could also vary in prevalence of other severe chronic illnesses [45].

Further detailed study of the reported severe manifestations might shed some light on pathogenesis. In Bikaner, where severe vivax malaria is commonly reported, high rates of acute kidney injury are reported in severe vivax malaria in children aged 0–5 years (8/41: 19.5%), an age group in whom acute tubular necrosis (the pathology implicated in falciparum malaria associated renal failure) is rare. Acidosis was reported in 7/65 children with vivax malaria (mean arterial pH 7.1) but its aetiology was unclear. In falciparum malaria acidosis is associated with a high lactate-pyruvate ratio indicating an anoxic pathogenesis, whereas in sepsis lactic pyruvate rations are not elevated. There is clearly a need for more in-depth prospective epidemiological studies, where background age-stratified population *P. vivax* parasitaemia prevalence is documented and there is proper evaluation of other concomitant illnesses. One such study from Papua, Indonesia, comparing population prevalence with coma in hospitalized patients estimated the incidence of cerebral malaria as 1:29,486 in *P. vivax* compared to 1:1,276 in *P. falciparum* infections. This suggested that *P. vivax*-associated coma is 23 times less common than *P. falciparum*-associated coma [18].

### Are *P. vivax*strains associated with severe disease intrinsically more pathogenic?

Epidemics of severe vivax malaria were reported from the USSR over fifty years ago. Changes in strain virulence could explain the recent and geographical restricted increase in reported cases, although there are no hard data available to support this hypothesis. Vivax strains clearly do differ between regions, which is most evident in the observed relapse patterns. Pathophysiological studies exploring difference in parasite virulence could reveal whether more virulent strains of this parasite with a once benign reputation have appeared.

An earlier systematic review on complicated vivax malaria in South America was reported by Lacerda and colleagues in 2012 [46]. In this review, most of the information derived from non-peer reviewed sources (including masters’ dissertations, doctoral theses, and national congresses’ abstracts) and all data came from the Brazilian literature [46]. A recent review by Baird included all published literature on all types of studies, including case reports (since 1990) and case series [47].

This analysis had several limitations. The pooled prevalence estimates of severe manifestations are based only on reported studies which include cases of severe vivax malaria and this provides a selection bias. Absence of severe disease amongst patients with vivax malaria appears much less likely to be reported. Assessing the pooled prevalence of severe vivax malaria against the general population or all vivax malaria cases as denominator is not feasible with the current data. Nearly all the estimated worldwide 390 million clinical cases of vivax malaria/year [4] are not reported in the studies. Except for one community-based study [14], all of the studies included in this systematic review are hospital-based, excluding case reports or small case series. As a consequence, the estimated pooled prevalence of the different severity manifestations represents proportions amongst patients considered sufficiently ill to warrant hospital admission. In addition, no definite criteria for the diagnosis of severe vivax malaria currently exist (they are “borrowed” from falciparum malaria except for the addition of thrombocytopenia, which has been inadequately justified), and many of the studies did not exclude possible co-morbidities or possible mixed *Plasmodium* infection. Another shortcoming is that we only selected English language articles, whereas there is a significant literature in other languages, e.g. reports from Amazonia are often in Portuguese.

In summary, there has been a marked increase in reported cases of severe vivax in certain geographical regions of the *P. vivax* endemic world, which cannot be explained with the current understanding of the disease and warrants further study into its aetiology. The use of thrombocytopenia as an independent severity criterion requires justification. In addition, more detailed epidemiological studies are needed, which adequately exclude co-morbidities, and take into account transmission intensity and anti-malarial drug susceptibility. As for severe *P. falciparum malaria*, *P. vivax-*specific criteria for defining severe disease are necessary to facilitate comparison between studies and establish its true prevalence.

## Electronic supplementary material

Additional file 1: **Variables, search terms, and search strategy used in this study.** (DOCX 24 KB)

Additional file 2: **Definitions for the diagnosis of severe vivax malaria in this study.** (DOCX 24 KB)

Additional file 3: **Characteristics of the 77 studies with reported severe vivax malaria patients.** (DOCX 97 KB)

Additional file 4: **Detailed analysis of pooled prevalence of severity signs among reported severe vivax malaria patients (77 studies).** (DOCX 38 KB)

Additional file 5: **Prevalence of cerebral malaria among both outpatients and inpatients of vivax malaria.** (DOCX 51 KB)

Additional file 6: **Prevalence of repeated generalized seizures among both outpatients and inpatients of vivax malaria.** (DOCX 27 KB)

Additional file 7: **Prevalence of renal dysfunction among both outpatients and inpatients of vivax malaria.** (DOCX 39 KB)

Additional file 8: **Prevalence of respiratory dysfunction among both outpatients and inpatients of vivax malaria.** (DOCX 40 KB)

Additional file 9: **Prevalence of hepatic dysfunction among both outpatients and inpatients of vivax malaria.** (DOCX 51 KB)

Additional file 10: **Prevalence of abnormal bleeding/DIC among both outpatients and inpatients of vivax malaria.** (DOCX 39 KB)

Additional file 11: **Prevalence of haemoglobinuria among both outpatients and inpatients of vivax malaria.** (DOCX 29 KB)

Additional file 12: **Prevalence of hypoglycaemia among both outpatients and inpatients of vivax malaria.** (DOCX 30 KB)

Additional file 13: **Prevalence of metabolic acidosis among both outpatients and inpatients of vivax malaria.** (DOCX 28 KB)

Additional file 14: **Prevalence of shock among both outpatients and inpatients of vivax malaria.** (DOCX 29 KB)

Additional file 15: **Prevalence of severe anaemia among both outpatients and inpatients of vivax malaria.** (DOCX 219 KB)

Additional file 16: **Prevalence of severe thrombocytopenia among both outpatients and inpatients of vivax malaria.** (DOCX 40 KB)

Additional file 17: **Prevalence of death among both outpatients and inpatients of vivax malaria.** (DOCX 52 KB)

Additional file 18: **Pooled prevalence of severity signs among both inpatients and outpatients of vivax malaria (62 studies).** (DOCX 24 KB)

Additional file 19: **Prevalence of cerebral malaria among only inpatients of vivax malaria.** (DOCX 34 KB)

Additional file 20: **Prevalence of repeated generalized seizures among only inpatients of vivax malaria.** (DOCX 29 KB)

Additional file 21: **Prevalence of renal dysfunction among only inpatients of vivax malaria.** (DOCX 29 KB)

Additional file 22: **Prevalence of respiratory dysfunction among only inpatients of vivax malaria.** (DOCX 32 KB)

Additional file 23: **Prevalence of hepatic dysfunction among only inpatients of vivax malaria.** (DOCX 36 KB)

Additional file 24: **Prevalence of abnormal bleeding/DIC among only inpatients of vivax malaria.** (DOCX 31 KB)

Additional file 25: **Prevalence of haemoglobinuria among only inpatients of vivax malaria.** (DOCX 26 KB)

Additional file 26: **Prevalence of hypoglycaemia among only inpatients of vivax malaria.** (DOCX 29 KB)

Additional file 27: **Prevalence of metabolic acidosis among only inpatients of vivax malaria.** (DOCX 28 KB)

Additional file 28: **Prevalence of shock among only inpatients of vivax malaria.** (DOCX 29 KB)

Additional file 29: **Prevalence of severe anaemia among only inpatients of vivax malaria.** (DOCX 34 KB)

Additional file 30: **Prevalence of severe thrombocytopenia among only inpatients of vivax malaria.** (DOCX 29 KB)

Additional file 31: **Prevalence of death among only inpatients of vivax malaria.** (DOCX 34 KB)

Additional file 32: **Pooled prevalence of severity signs among only inpatients of vivax malaria (15 studies).** (DOCX 24 KB)

## Abbreviations

AFRO: Africa
AMRO: Americas
EMRO: Eastern Mediterranean
EURO: Europe
SEARO: South-East Asia
WPRO: Western Pacific
DIC: Disseminated intravascular coagulation
MOD: Multi-organ dysfunction
PBS: Peripheral blood smear
RDT: Rapid diagnostic test.
