# Supplementary material for: Severe vivax malaria: a systematic review and meta-analysis of clinical studies since 1900
Source: Malar J. 2014 Dec 8;13:481. doi: 10.1186/1475-2875-13-481 (PMC4364574; doi:10.1186/1475-2875-13-481)
Supplement: Supplementary file 1 — Additional file 1: Variables, search terms, and search strategy used in this study. (DOCX 24 KB) [file 12936_2014_3678_MOESM1_ESM.docx]

**Additional file 1. Variables, search terms, and search strategy used in this study**

| Variables | Total number of patients with vivax malaria, number of patients with severe vivax malaria, location (country), region, nationality, malaria endemicity, age (years), gender, PBS, parasitaemia (/mm^3^), PCR confirmation, RDT, pregnancy, duration of illness, duration of hospitalization, antibiotics used, neurologic dysfunction, psychological dysfunction, cardiologic dysfunction, respiratory dysfunction, renal dysfunction, GI dysfunction, liver dysfunction, acidosis, hypoglycaemia, coagulopathy and bleeding, circulatory collapse or shock, hepatomegaly, splenomegaly, thrombocyte count (/mm^3^), Hb, TLC, ICU admission, multi-organ dysfunction, anti-malarial drug(s) used, other unusual manifestations, death, co-morbidity, underlying diseases. |
| --- | --- |
| Search terms | **Patients:** (1) *Plasmodium vivax*, (2) Severe OR complicated, (3) 1 AND 2.  **Outcomes: (**4) Cerebral complication, (5) Convulsions, (6) Kidney injury, (7) Renal failure, (8) Hemoglobinuria, (9) Circulatory collapse, (10) Shock, (11) Jaundice, (12) Hyperbilirubinemia, (13) Hepatic dysfunction, (14) Bleeding, (15) Hemorrhage, (16) Thrombocytopenia, (17) Disseminated intravascular coagulation, (18) DIC, (19) Acute respiratory distress syndrome, (20) ARDS, (21) Pulmonary edema, (22) Metabolic acidosis, (23) Hyperlactatemia, (24) Severe anemia, (25) Hypoglycemia, (26) Death |
| Search strategy | **Medline (PubMed):** (27) 4 OR 5 OR 6 OR 7 OR 8 OR 9 OR 10 OR 11 OR 12 OR 13 OR 14 OR 15 OR 16 OR 17 OR 18 OR 19 OR 20 OR 21 OR 22 OR 23 OR 24 OR 25 OR 26, (28) 3 AND 27.  **Scopus (Scopus):** (27) 4 OR 5, (28) 6 OR 7 OR 8, (29) 9 OR 10, (30) 11 OR 12 OR 13, (31) 14 OR 15 OR 16, (32) 17 OR 18, (33) 19 OR 20 OR 21, (34) 27 OR 28 OR 29 OR 30 OR 31 OR 32 OR 33, (35) 34 OR 22 OR 23 OR 24 OR 25 OR 26, (36) 3 AND 35. |
