# Supplementary material for: Severe vivax malaria: a systematic review and meta-analysis of clinical studies since 1900
Source: Malar J. 2014 Dec 8;13:481. doi: 10.1186/1475-2875-13-481 (PMC4364574; doi:10.1186/1475-2875-13-481)
Supplement: Supplementary file 2 — Additional file 2: Definitions for the diagnosis of severe vivax malaria in this study. (DOCX 24 KB) [file 12936_2014_3678_MOESM2_ESM.docx]

**Additional file 2. Definitions for the diagnosis of severe vivax malaria in this study**

| **Severity sign** | **Definition** |
| --- | --- |
| Cerebral malaria | Unrousable coma (GCS* ≤9), impaired consciousness, prostration, i.e. generalized weakness so that the patient is unable to walk, or sit up without assistance |
| Repeated generalized seizures | ≥ 2 seizures observed within 24 hours |
| Renal dysfunction | Serum creatinine >3 mg/dL |
| Respiratory dysfunction | Pulmonary oedema (radiological), ALI* (PaO_2_:FiO_2_<300 mmHg), ARDS*( PaO_2_:FiO_2_<200 mmHg), respiratory failure |
| Hepatic dysfunction | Jaundice, hyperbilirubinaemia (total bilirubin >3 mg/dl), AST/ALT values >3 times of normal |
| Abnormal bleeding or DIC | Spontaneous abnormal bleeding or laboratory evidence of DIC |
| Haemoglobinuria | Haemolysis (not secondary to G6PD deficiency) and laboratory evidence of haemoglobinuria |
| Hypoglycaemia | Blood glucose <40 mg/dL (<2.22 mmol/L) |
| Metabolic acidosis | Arterial pH <7.25 or plasma bicarbonate <15 mmol/L |
| Hyperlactataemia | Blood lactate > 5 mmol/L |
| Circulatory collapse/shock | Blood pressure <70 mmHg in >5-year-old children and adult, <50 mmHg in ≤5-year-old children |
| Severe anaemia | Haemoglobin <5 g/dL |
| Severe thrombocytopaenia | Platelet count <50,000/mm^3^ |

*ALI = Acute Lung Injury, ARDS = Acute Respiratory Distress Syndrome, GCS = Glasgow Coma Score
