# Supplementary material for: Severe vivax malaria: a systematic review and meta-analysis of clinical studies since 1900
Source: Malar J. 2014 Dec 8;13:481. doi: 10.1186/1475-2875-13-481 (PMC4364574; doi:10.1186/1475-2875-13-481)
Supplement: Supplementary file 3 — Additional file 3: Characteristics of the 77 studies with reported severe vivax malaria patients. (DOCX 97 KB) [file 12936_2014_3678_MOESM3_ESM.docx]

**Additional file 3. Characteristics of the 77 studies with reported severe vivax malaria patients**

| **Author** | **Publication Year** | **WHO region** | **Location** | **Type of cases §** | **PCR confirmation** | **Type of patients** | **Age, years^a^** | **Gender (Male/Female), %^b^** | **Co-morbidities rule out?** |
| --- | --- | --- | --- | --- | --- | --- | --- | --- | --- |
| Bahr [[18](#_ENREF_18)] | 1928 | AMRO | USA | Both | No | Adults** | 55** | 66.7/33.3** | No |
| Giglioli[[19](#_ENREF_19)] | 1930 | AMRO | British Guiana | Inpts | No | NA | NA | NA | No |
| Fitz-Hugh [[20](#_ENREF_20)] | 1944 | SEARO | India | Both | No | NA | NA | NA | No |
| Horn [[21](#_ENREF_21)] | 1944 | AMRO | USA^c^ | Both | No | NA | NA | NA | No |
| Read [[22](#_ENREF_22)] | 1946 | AMRO | USA | Both | No | NA | NA | NA | No |
| Lippincott [[23](#_ENREF_23)] | 1946 | AMRO | USA | Both | No | NA | NA | NA | No |
| Whorton[[24](#_ENREF_24)] | 1947 | AMRO | USA | Both | No | NA | NA | NA | No |
| Martelo[[25](#_ENREF_25)] | 1969 | AMRO | USA^d^ | Both | No | NA | NA | NA | No |
| Gopinathan[[26](#_ENREF_26)] | 1982 | SEARO | India | Both | No | Adults | 34 | 100/0 | Yes |
| Lynk[[27](#_ENREF_27)] | 1989 | AMRO | Canada ^e^ | Both | No | Children | NA | NA | No |
| Singh [[28](#_ENREF_28)] | 1992 | SEARO | India | Both | No | Both | NA | 50.5/49.5 | No |
| Wattanagoon[[29](#_ENREF_29)] | 1994 | SEARO | Thailand | Both | No | Children | NA | NA | No |
| Svenson[[30](#_ENREF_30)] | 1995 | AMRO | Canada ^e^ | Both | No | Adults | 33.4 | 66/34 | No |
| Luxemburger[[31](#_ENREF_31)] | 1997 | SEARO | Thailand | Both | No | Both | 2.1 | 50/50 | No |
| Lee [[32](#_ENREF_32)] | 1997 | SEARO | Thailand | Both | No | Adults | 24.5 (median) | 29.2/70.8 | No |
| Basu[[33](#_ENREF_33)] | 1998 | SEARO | India | Both | No | Children | NA | NA | No |
| McNeeley[[34](#_ENREF_34)] | 1998 | AMRO | USA ^e^ | Both | No | Both | 31 (median) | 69/31 | No |
| Oh [[35](#_ENREF_35)] | 2001 | WPRO | South Korea | Both | No | Adults | 23 (median) | 93:8 (ratio) | No |
| Mehta [[36](#_ENREF_36)] | 2001 | SEARO | India | Both | No | Adults | NA | 79.2/20.8 | Yes |
| Mohapatra[[37](#_ENREF_37)] | 2002 | SEARO | India | Both | No | Adults | 15-60 (range) | 63.6/36.4 | No |
| Song [[38](#_ENREF_38)] | 2003 | WPRO | South Korea | Both | No | Adults | 35 | 75/25 | No |
| Vicas[[39](#_ENREF_39)] | 2005 | AMRO | USA^e^ | Both | No | Both | 26.6 | 55.6/44.4 | No |
| Rodriguez-Morales [[40](#_ENREF_40)] | 2006 | AMRO | Venezuela | Both | No | Children | 3.97 | 47.4/52.6 | No |
| Barcus[[12](#_ENREF_12)] | 2007 | SEARO | Indonesia | Both | No | Both | 6 (in children), 30 (in adults) | Ratio 1.29 (children), 1.31 (adults) | No |
| Genton[[14](#_ENREF_14)] | 2008 | WPRO | PNG | Both | No | Children | NA | NA | No |
| Tjitra[[13](#_ENREF_13)] | 2008 | SEARO | Indonesia | Both | No | Both | NA | NA | No |
| Beg [[41](#_ENREF_41)] | 2008 | EMRO | Pakistan | Both | Yes | Both | 34.9 | 60.4/39.6 | No |
| Nayak[[42](#_ENREF_42)] | 2009 | SEARO | India | Both | No | Adults | NA | 0/100 | Yes |
| Poespoprodjo[[43](#_ENREF_43)] | 2009 | SEARO | Indonesia | Both | No | Children | NA | NA | Yes |
| Khan [[44](#_ENREF_44)] | 2009 | EMRO | Qatar^e^ | Both | No | Both | 26 (median) | 79/21 | No |
| Sharma [[45](#_ENREF_45)] | 2009 | SEARO | India | Both | No | Both | 32 (median) | 72.8/27.2 | No |
| Rodriguez-Morales [[46](#_ENREF_46)] | 2009 | AMRO | Venezuela | Inpts | No | Both | 27 | 64.7/35.3 | No |
| Kochar[[47](#_ENREF_47)] | 2009 | SEARO | India | Both | Yes | Adults | 29.65** | 72.5/27.5 | Yes |
| Kochar[[48](#_ENREF_48)] | 2010 | SEARO | India | Both | Yes | Children | NA | 67/33 | Yes |
| Andrade [[49](#_ENREF_49)] | 2010 | AMRO | Brazil | Both | Yes | Both | Median 42 (in 60), 33 (in 50), 22 (in 19) | 48/52 | Yes |
| George [[50](#_ENREF_50)] | 2010 | SEARO | India | Inpts | No | Adults | 42 (median) | NA | Yes |
| Manning [[51](#_ENREF_51)] | 2011 | WPRO | PNG | Inpts | Yes | Children | 2.75 (median) | 55.6/44.4 | Yes |
| Nayak[[52](#_ENREF_52)] | 2011 | SEARO | India | Inpts | No | Adults | NA | 72.5/27.5 | Yes |
| Franklin [[53](#_ENREF_53)] | 2011 | AMRO | Brazil | Both | Yes | Adults | 49 (median) | 61.9/38.1 | Yes |
| Mitja[[54](#_ENREF_54)] | 2011 | WPRO | PNG | Both | No | Both | 7.6 | 71.4*/28.6* | No |
| Deepa[[55](#_ENREF_55)] | 2011 | SEARO | India | Both | No | Both | 1-71 (range) | 8:2 (ratio) | No |
| Murgod[[56](#_ENREF_56)] | 2011 | SEARO | India | Both | No | Both | 2-57 (range) | 81.4/18.6 | Yes |
| Lampah[[57](#_ENREF_57)] | 2011 | SEARO | Indonesia | Both | Yes | Both** | 19 (median)** | 66.7/33.3** | Yes |
| Srivastava [[58](#_ENREF_58)] | 2011 | SEARO | India | Both | No | Adults | 38.5 | 60/40 | No |
| Singh [[59](#_ENREF_59)] | 2011 | SEARO | India | Both | No | Children | 5.4 | 56.5/43.5 | No |
| Garg [[60](#_ENREF_60)] | 2012 | SEARO | India | Both | Yes | Both** | 30.172** | 56.7/43.3** | Yes |
| Mahgoub[[61](#_ENREF_61)] | 2012 | EMRO | Sudan | Inpts | No | Children | 4.9 | 55.6/44.4 | No |
| Kaushik [[62](#_ENREF_62)] | 2012 | SEARO | India | Both | No | Children | 0.66-12 (range) | 75/25 | Yes |
| Nadkar[[63](#_ENREF_63)] | 2012 | SEARO | India | Inpts | No | NA | 51.45 (in dead), 41.38 (in survived) | 71.9/28.1 | No |
| Tanwar[[64](#_ENREF_64)] | 2012 | SEARO | India | Both | Yes | Children | NA | 69.8/30.2 | Yes |
| Yadav [[65](#_ENREF_65)] | 2012 | SEARO | India | Inpts | No | Children | 3 (median) | 74.8/25.2 | No |
| Shaikh [[66](#_ENREF_66)] | 2012 | EMRO | Pakistan | Both | Yes | Children | NA | 53.3/46.7 | No |
| Lanca[[67](#_ENREF_67)] | 2012 | AMRO | Brazil | Inpts | No | Children | 0-14 (range) | 70.8/29.2 | Yes |
| Mehmood[[68](#_ENREF_68)] | 2012 | EMRO | Pakistan | Both | No | NA | 41 (median) | 80.4/19.6 | No |
| Naha [[15](#_ENREF_15)] | 2012 | SEARO | India | Both | No | Adults | 33.17 | 80.7/19.3 | No |
| Sharma [[69](#_ENREF_69)] | 2012 | SEARO | India | Both | No | Children | 5.025 | 75.2/24.8 | No |
| Nandwani[[70](#_ENREF_70)] | 2012 | SEARO | India | Inpts | No | Adults | 34.85 | 55/45 | Yes |
| Limaye[[16](#_ENREF_16)] | 2012 | SEARO | India | Both | No | Adults | 29 (median) | NA | No |
| Nurleila[[71](#_ENREF_71)] | 2012 | SEARO | Indonesia | Both | No | Both | NA | 45.5/54.5 | No |
| Barber [[72](#_ENREF_72)] | 2013 | WPRO | Malaysia | Both | Yes | Both | 24 (median) | 77/23 | Yes |
| Singh [[73](#_ENREF_73)] | 2013 | SEARO | India | Both | No | Children | 12 (median) | 68.8/31.2 | No |
| Zaki[[74](#_ENREF_74)] | 2013 | SEARO | India | Both | No | Children | NA | 66.7/33.3 | Yes |
| Douglas [[75](#_ENREF_75)] | 2013 | SEARO | Indonesia | Both | No | Both | NA | NA | No |
| Lon [[76](#_ENREF_76)] | 2013 | WPRO | Cambodia | Inpts | No | Both | 18 (median) | 57/43 | No |
| Abdallah [[77](#_ENREF_77)] | 2013 | EMRO | Sudan | Inpts | No | Adults | 34.2 | NA | No |
| Sharma [[78](#_ENREF_78)] | 2013 | SEARO | India | Inpts | No | Children | 6.7 | 61/39 | No |
| Gehlawat[[79](#_ENREF_79)] | 2013 | SEARO | India | Inpts | No | Children | NA | NA |  |
| Leal-Santos [[80](#_ENREF_80)] | 2013 | AMRO | Brazil | Both | No | Both | 37.7 | 78/22 | No |
| Raza [[81](#_ENREF_81)] | 2013 | EMRO | Pakistan | Both | Yes | Adults | 42 (median) | NA | Yes |
| Bhatacharjee[[82](#_ENREF_82)] | 2013 | SEARO | India | Both | No | Children | NA | 54.2/45.8 | Yes |
| Ketema[[83](#_ENREF_83)] | 2013 | EMRO | Ethiopia | Both | No | Children | 4.25 (median) | 57.5/42.5 | No |
| Sarkar [[84](#_ENREF_84)] | 2013 | SEARO | India | Both | No | Adults | NA | NA | No |
| Zubairi[[85](#_ENREF_85)] | 2013 | EMRO | Pakistan | Inpts | Yes | Adults | 42 | 66.9/33.1 | Yes |
| Aatif[[86](#_ENREF_86)] | 2013 | EMRO | Pakistan | Both | No | Adults | 30.21 | 67.3/32.7 | No |
| Rizvi [[87](#_ENREF_87)] | 2013 | SEARO | India | Both | No | Adults | 31.11 | 59.7/40.3 | No |
| Kwak[[88](#_ENREF_88)] | 2013 | WPRO | South Korea | Both | No | Adults | 42.5 | 69/31 | No |
| Jain [[89](#_ENREF_89)] | 2013 | SEARO | India | Both | Yes | Both | NA | NA |  |

* In this study Mean and SD are of 232/1213 patients, while gender (5 and 2) are of only 7 severe vivax patients.

** This is data of only severe vivax cases. Data of others is unavailable.

^a^Age is given in mean, unless otherwise mentioned

**^b^** It is in %, unless otherwise mentioned as male:female ratio

**^c^**Vivax malaria infection was acquired in Sicily and Eastern Base Section

^d^Vivax malaria infection was acquired in Vietnam and USA

^e^Vivax malaria infection was acquired in different endemic countries

**§** Both = Both inpatients and outpatients of vivax malaria Inpts = Only inpatients of vivax malaria
