# Supplementary material for: Severe vivax malaria: a systematic review and meta-analysis of clinical studies since 1900
Source: Malar J. 2014 Dec 8;13:481. doi: 10.1186/1475-2875-13-481 (PMC4364574; doi:10.1186/1475-2875-13-481)
Supplement: Supplementary file 4 — Additional file 4: Detailed analysis of pooled prevalence of severity signs among reported severe vivax malaria patients (77 studies). (DOCX 38 KB) [file 12936_2014_3678_MOESM4_ESM.docx]

**Additional file 4. Detailed analysis of pooled prevalence of severity signs among reported severe vivax malaria patients (77 studies)**

| **Severity sign** | **No. of studies** | **No. of events** | **No. of patients** | **Prevalence** | **95% CI** | **p-value** |
| --- | --- | --- | --- | --- | --- | --- |
| Severe thrombocytopaenia | 27 | 888 | 45775 | 8.6 | 5.4–11.8 |  |
| Since 2000 | 25 | 883 | 39174 | 10.8 | 7.1–14.5 |  |
| WHO region |  |  |  |  |  |  |
| SEARO | 14 | 443 | 38302 | 8.2 | 3.1–13.3 | <0.001 |
| AMRO | 3 | 40 | 2310 | 4.7 | 1.2–9.1 |  |
| EMRO | 5 | 221 | 1404 | 18.6 | 0–41.1 |  |
| WPRO | 5 | 184 | 3759 | 13.3 | 0–26.8 |  |
| Country |  |  |  |  |  |  |
| India | 12 | 417 | 6933 | 9.5 | 3.6–15.3 | <0.001 |
| Pakistan | 4 | 217 | 1182 | 27.3 | 0–56.9 |  |
| South Korea | 3 | 162 | 497 | 22.5 | 0–49.5 |  |
| Metabolic acidosis | 8 | 138 | 46341 | 1 | 0–2.3 |  |
| Since 2000 | 8 | 138 | 39740 | 1.3 | 0–2.9 |  |
| SEARO | 4 | 88 | 38868 | 1.1 | 0–3.1 |  |
| India | 3 | 85 | 6933 | 1.8 | 0–6 |  |
| Renal dysfunction | 20 | 244 | 46341 | 1.1 | 0.3–1.9 |  |
| Since 2000 | 20 | 244 | 39740 | 1.5 | 0.6–2.4 |  |
| SEARO | 17 | 231 | 38868 | 2 | 0.9–3.1 |  |
| India | 16 | 227 | 6933 | 2.9 | 1.3–4.6 |  |
| Shock | 77 | 67 | 46411 | 5.1 | 2.5–7.7 |  |
| Since 2000 | 60 | 67 | 39740 | 5.1 | 2.5–7.7 |  |
| SEARO | 42 | 37 | 38868 | 3.7 | 2.1–6.3 |  |
| India | 33 | 37 | 6933 | 3.7 | 1.2–6.3 |  |
| Hepatic dysfunction | 77 | 727 | 46536 | 4.2 | 3.2–5.2 |  |
| Period |  |  |  |  |  |  |
| Before 2000 | 17 | 37 | 6796 | 0.6 | 0–3.1 | <0.001 |
| Since 2000 | 60 | 690 | 39740 | 5.6 | 4.3–6.9 |  |
| WHO region |  |  |  |  |  |  |
| SEARO | 42 | 571 | 38868 | 7 | 4.7–9.2 | <0.001 |
| AMRO | 17 | 56 | 2505 | 1.6 | 0.1–3.1 |  |
| EMRO | 10 | 88 | 1404 | 5.8 | 2.3–9.4 |  |
| WPRO | 8 | 12 | 3759 | 3.7 | 0–9.2 |  |
| Country |  |  |  |  |  |  |
| India | 33 | 561 | 6933 | 9.4 | 6.6–12.2 | <0.001 |
| Pakistan | 6 | 78 | 1182 | 5.8 | 1.6–10.1 |  |
| USA | 8 | 36 | 1765 | 1.5 | 0–3.3 |  |
| Brazil | 4 | 14 | 360 | 4.6 | 0–9.8 |  |
| Severe anaemia | 77 | 2276 | 46411 | 4 | 2.9–5.1 |  |
| Since 2000 | 60 | 2275 | 39740 | 5.3 | 4.1–6.5 |  |
| WHO region |  |  |  |  |  |  |
| SEARO | 42 | 2204 | 38868 | 7.4 | 5.5–9.4 | <0.001 |
| AMRO | 17 | 13 | 2380 | 8.8 | 3.1–14.5 |  |
| EMRO | 10 | 25 | 1404 | 7.3 | 1.8–12.8 |  |
| WPRO | 8 | 34 | 3759 | 0.2 | 0–0.9 |  |
| Country |  |  |  |  |  |  |
| India | 33 | 372 | 6933 | 7.9 | 5.7–10.2 | <0.001 |
| Indonesia | 6 | 1832 | 28878 | 9 | 0–18.2 |  |
| PNG | 3 | 23 | 3186 | 0.6 | 0–1.3 |  |
| Hypoglycemia | 77 | 44 | 46381 | 1.8 | 0.9–2.7 |  |
| Since 2000 | 60 | 44 | 39710 | 1.8 | 0.9–2.7 |  |
| WHO region |  |  |  |  |  |  |
| SEARO | 42 | 24 | 38838 | 1.7 | 0.6–2.8 | <0.001 |
| EMRO | 10 | 17 | 1404 | 0.1 | 0–0.003 |  |
| India | 33 | 24 | 6903 | 5.7 | 5.3–6.1 |  |
| Cerebral malaria | 77 | 532 | 45845 | 0.8 | 0.5–1.1 |  |
| Period |  |  |  |  |  |  |
| Before 2000 | 17 | 14 | 6671 | 0.3 | 0–0.5 | <0.001 |
| Since 2000 | 60 | 518 | 39174 | 1.5 | 1–1.9 |  |
| WHO region |  |  |  |  |  |  |
| SEARO | 42 | 464 | 38302 | 1.5 | 1.1–2 | <0.001 |
| EMRO | 10 | 32 | 1404 | 1.6 | 0.1–3 |  |
| WPRO | 8 | 29 | 3759 | 0.8 | 0–2.5 |  |
| Country |  |  |  |  |  |  |
| India | 33 | 316 | 6933 | 3.6 | 2.3–4.8 | <0.001 |
| Indonesia | 6 | 145 | 28312 | 1.3 | 0.4–2.2 |  |
| Pakistan | 6 | 25 | 1182 | 1.5 | 0–3.4 |  |
| Abnormal bleeding/DIC | 77 | 206 | 46346 | 0.6 | 0.2–1 |  |
| Period |  |  |  |  |  |  |
| Before 2000 | 17 | 11 | 6601 | 1.8 | 0.7–2.8 | <0.001 |
| Since 2000 | 60 | 195 | 39745 | 0.9 | 0.4–1.4 |  |
| WHO region |  |  |  |  |  |  |
| SEARO | 42 | 161 | 38868 | 0.9 | 0.3–1.4 | <0.001 |
| AMRO | 17 | 13 | 2315 | 3.1 | 0.1–6.1 |  |
| EMRO | 10 | 26 | 1404 | 1.85 | 1.2–2.7 |  |
| India | 33 | 155 | 6933 | 1.7 | 0.6–2.8 |  |
| Respiratory dysfunction | 77 | 144 | 46411 | 0.3 | 0.1–0.5 |  |
| Since 2000 | 60 | 142 | 39740 | 0.6 | 0.2–0.9 |  |
| WHO region |  |  |  |  |  |  |
| SEARO | 42 | 76 | 39077 | 0.1 | 0–0.3 | <0.001 |
| AMRO | 17 | 11 | 2171 | 3 | 0–6.6 |  |
| EMRO | 10 | 56 | 1404 | 2 | 0–4.7 |  |
| Country |  |  |  |  |  |  |
| India | 33 | 76 | 7142 | 0.4 | 0.1–0.6 | <0.001 |
| Pakistan | 6 | 56 | 1182 | 3.5 | 0–7.2 |  |
| Death | 77 | 353 | 46411 | 0.3 | 0.1–0.4 |  |
| Period |  |  |  |  |  |  |
| Before 2000 | 17 | 11 | 6671 | 0.2 | 0–0.6 | <0.001 |
| Since 2000 | 60 | 342 | 39740 | 0.4 | 0.2–0.6 |  |
| WHO region |  |  |  |  |  |  |
| SEARO | 42 | 324 | 38868 | 0.4 | 0.2–0.7 | 0.242 |
| AMRO | 17 | 18 | 2380 | 6.1 | 0.2–3 |  |
| EMRO | 10 | 6 | 1404 | 0.7 | 0.3–1.1 |  |
| Country |  |  |  |  |  |  |
| USA | 8 | 5 | 1640 | 1.2 | 0–2.9 | <0.001 |
| India | 33 | 132 | 6933 | 0.9 | 0.4–1.5 |  |
| Pakistan | 6 | 6 | 1182 | 0.7 | 0.3–1.1 |  |
| Indonesia | 6 | 191 | 28878 | 0.7 | 0.4–1.1 |  |
| Multiple convulsions | 77 | 88 | 46386 | 0.2 | 0–0.5 |  |
| Since 2000 | 60 | 88 | 39715 | 0.4 | 0–0.9 |  |
| WHO region |  |  |  |  |  |  |
| SEARO | 42 | 54 | 38838 | 0.2 | 0–0.6 | <0.001 |
| EMRO | 10 | 31 | 1404 | 10.6 | 2.6–18.6 |  |
| India | 33 | 52 | 6903 | 5.1 | 1.8–8.4 |  |
| Haemoglobinuria | 77 | 93 | 46411 | 0.1 | 0–0.4 |  |
| Since 2000 | 60 | 93 | 39740 | 0.2 | 0–0.6 |  |
| SEARO | 42 | 23 | 38868 | 2.2 | 0.6–3.7 |  |
| India | 33 | 23 | 6933 | 2.2 | 0.6–3.7 |  |
