# Supplementary material for: Severe vivax malaria: a systematic review and meta-analysis of clinical studies since 1900
Source: Malar J. 2014 Dec 8;13:481. doi: 10.1186/1475-2875-13-481 (PMC4364574; doi:10.1186/1475-2875-13-481)
Supplement: Supplementary file 5 — Additional file 5: Prevalence of cerebral malaria among both outpatients and inpatients of vivax malaria. (DOCX 51 KB) [file 12936_2014_3678_MOESM5_ESM.docx]

**Additional file 5. Prevalence of cerebral malaria among both outpatients and inpatients of vivax malaria**

| **Author (Reference)** | **Year** | **Country** | **Study design** | **Total vivax** | **Cerebral malaria** | **Prevalence** | **95% CI** |
| --- | --- | --- | --- | --- | --- | --- | --- |
| Fitz-Hugh [[20](#_ENREF_20)] | 1944 | India | RHBS | 1375 | 5 | 0.4 | 0.1–0.8 |
| Horn [[21](#_ENREF_21)] | 1944 | USA | RHBS | 452 | 2 | 0.4 | 0.05–1.6 |
| Gopinathan[[26](#_ENREF_26)] | 1982 | India | RHBS | 178 | 2 | 1.1 | 0.1–4.0 |
| Luxemburger[[31](#_ENREF_31)] | 1997 | Thailand | PHBS | 2573 | 3 | 0.1 | 0.02–0.3 |
| Basu[[33](#_ENREF_33)] | 1998 | India | PHBS | 141 | 2 | 1.4 | 0.2–5.0 |
| Mohapatra[[37](#_ENREF_37)] | 2002 | India | PHBS | 110 | 1 | 0.9 | 0.02–5.0 |
| Barcus[[12](#_ENREF_12)] | 2007 | Indonesia | RHBS | 1135 | 3 | 0.3 | 0.05–0.8 |
| Genton[[14](#_ENREF_14)] | 2008 | PNG | PHBS | 1946 | 3 | 0.1 | 0.03–0.4 |
| Tjitra[[13](#_ENREF_13)] | 2008 | Indonesia | PHBS | 2937 | 41 | 1.4 | 1.0–1.9 |
| Poespoprodjo[[43](#_ENREF_43)] | 2009 | Indonesia | PHBS | 102 | 2 | 2.0 | 0.2–6.9 |
| Kochar[[47](#_ENREF_47)] | 2009 | India | PHBS | 456 | 5 | 1.1 | 0.4–2.5 |
| Khan [[44](#_ENREF_44)] | 2009 | Qatar | PHBS | 39 | 2 | 5.1 | 0.6–17.3 |
| Kochar[[48](#_ENREF_48)] | 2010 | India | PHBS | 103 | 9 | 8.7 | 4.1–15.9 |
| Srivastava [[58](#_ENREF_58)] | 2011 | India | RHBS | 50 | 2 | 4.0 | 0.5–13.7 |
| Singh [[59](#_ENREF_59)] | 2011 | India | RHBS | 108 | 3 | 2.8 | 0.6–8.0 |
| Lampah[[57](#_ENREF_57)] | 2011 | Indonesia | PHBS | 2443 | 6 | 0.2 | 0.1–0.5 |
| Kaushik [[62](#_ENREF_62)] | 2012 | India | PHBS | 35 | 12 | 34.3 | 19.1–52.2 |
| Shaikh [[66](#_ENREF_66)] | 2012 | Pakistan | RHBS | 192 | 17 | 8.8 | 5.2–13.8 |
| Mehmood[[68](#_ENREF_68)] | 2012 | Pakistan | RHBS | 97 | 2 | 2.1 | 0.2–7.2 |
| Naha [[15](#_ENREF_15)] | 2012 | India | RHBS | 213 | 3 | 1.4 | 0.3–4.1 |
| Sharma [[69](#_ENREF_69)] | 2012 | India | RHBS | 105 | 20 | 19.0 | 12.0–27.9 |
| Limaye[[16](#_ENREF_16)] | 2012 | India | RHBS | 338 | 12 | 3.5 | 1.8–6.1 |
| Nurleila[[71](#_ENREF_71)] | 2012 | Indonesia | RHBS | 1837 | 93 | 5.1 | 4.1–6.2 |
| Garg [[60](#_ENREF_60)] | 2012 | India | PHBS | 78 | 7 | 9.0 | 3.7–17.6 |
| Singh[[73](#_ENREF_73)] | 2013 | India | PHBS | 61 | 10 | 16.4 | 8.1–28.09 |
| Zaki[[74](#_ENREF_74)] | 2013 | India | RHBS | 133 | 1 | 0.75 | 0.02–4.12 |
| Bhatacharjee[[82](#_ENREF_82)] | 2013 | India | RHBS | 168 | 10 | 5.95 | 2.89–10.67 |
| Sarkar [[84](#_ENREF_84)] | 2013 | India | PHBS | 900 | 112 | 12.44 | 10.36–14.78 |
| Rizvi [[87](#_ENREF_87)] | 2013 | India | RHBS | 172 | 10 | 5.81 | 2.82–10.43 |
| Kwak[[88](#_ENREF_88)] | 2013 | South Korea | RHBS | 352 | 3 | 0.85 | 0.18–2.47 |
| Jain [[89](#_ENREF_89)] | 2013 | India | PHBS | 198 | 7 | 3.53 | 1.43–7.15 |
| Pooled |  |  |  | 44478 | 410 | 0.6 | 0.3–0.8 |

PHBS = Prospective hospital-based study

PCBS = Prospective community-based study (conducted in health sub-center)

RHBS = Retrospective hospital-based study

PNG = Papua New Guinea
