# Supplementary material for: Severe vivax malaria: a systematic review and meta-analysis of clinical studies since 1900
Source: Malar J. 2014 Dec 8;13:481. doi: 10.1186/1475-2875-13-481 (PMC4364574; doi:10.1186/1475-2875-13-481)
Supplement: Supplementary file 6 — Additional file 6: Prevalence of repeated generalized seizures among both outpatients and inpatients of vivax malaria. (DOCX 27 KB) [file 12936_2014_3678_MOESM6_ESM.docx]

**Additional file 6. Prevalence of repeated generalized seizures among both outpatients and inpatients of vivax malaria**

| **Author (Reference)** | **Year** | **Country** | **Study design** | **Total vivax** | **Repeated generalized seizures** | **Prevalence** | **95% CI** |
| --- | --- | --- | --- | --- | --- | --- | --- |
| Barcus[[12](#_ENREF_12)] | 2007 | Indonesia | RHBS | 1135 | 2 | 0.2 | 0.02–0.6 |
| Singh [[59](#_ENREF_59)] | 2011 | India | RHBS | 108 | 4 | 3.7 | 1.0–9.2 |
| Shaikh [[66](#_ENREF_66)] | 2012 | Pakistan | RHBS | 192 | 27 | 14.1 | 9.5–19.8 |
| Barber [[72](#_ENREF_72)] | 2013 | Malaysia | PHBS | 43 | 1 | 2.3 | 0.1–12.3 |
| Bhatacharjee[[82](#_ENREF_82)] | 2013 | India | RHBS | 168 | 8 | 4.76 | 2.08–9.17 |
| Sarkar [[84](#_ENREF_84)] | 2013 | India | PHBS | 900 | 28 | 3.11 | 2.08–4.46 |
| Pooled |  |  |  | 45014 | 70 | 0.1 | 0–0.3 |
