# Supplementary material for: Severe vivax malaria: a systematic review and meta-analysis of clinical studies since 1900
Source: Malar J. 2014 Dec 8;13:481. doi: 10.1186/1475-2875-13-481 (PMC4364574; doi:10.1186/1475-2875-13-481)
Supplement: Supplementary file 7 — Additional file 7: Prevalence of renal dysfunction among both outpatients and inpatients of vivax malaria. (DOCX 39 KB) [file 12936_2014_3678_MOESM7_ESM.docx]

**Additional file 7. Prevalence of renal dysfunction among both outpatients and inpatients of vivax malaria**

| **Author (Reference)** | **Year** | **Country** | **Study design** | **Total vivax** | **Renal dysfunction** | **Prevalence** | **95% CI** |
| --- | --- | --- | --- | --- | --- | --- | --- |
| Mehta [[36](#_ENREF_36)] | 2001 | India | RHBS | 219 | 3 | 1.4 | 0.3–3.9 |
| Barcus[[12](#_ENREF_12)] | 2007 | Indonesia | RHBS | 1135 | 4 | 0.3 | 0.1–0.9 |
| Kochar[[47](#_ENREF_47)] | 2009 | India | PHBS | 456 | 18 | 3.9 | 2.3–6.2 |
| Nayak[[42](#_ENREF_42)] | 2009 | India | PHBS | 169 | 7 | 4.1 | 1.7–8.3 |
| Sharma [[45](#_ENREF_45)] | 2009 | India | RHBS | 221 | 1 | 0.4 | 0.01–2.5 |
| Kochar[[48](#_ENREF_48)] | 2010 | India | PHBS | 103 | 10 | 9.7 | 4.7–17.1 |
| Singh [[59](#_ENREF_59)] | 2011 | India | RHBS | 108 | 6 | 5.6 | 2.1–11.7 |
| Naha [[15](#_ENREF_15)] | 2012 | India | RHBS | 213 | 2 | 0.9 | 0.1–3.3 |
| Sharma [[69](#_ENREF_69)] | 2012 | India | RHBS | 105 | 5 | 4.8 | 1.6–10.8 |
| Limaye[[16](#_ENREF_16)] | 2012 | India | RHBS | 338 | 12 | 3.5 | 1.8–6.1 |
| Garg [[60](#_ENREF_60)] | 2012 | India | PHBS | 78 | 3 | 3.8 | 0.8–10.8 |
| Naha [[15](#_ENREF_15)] | 2012 | India | RHBS | 213 | 2 | 0.9 | 0.1–3.3 |
| Zaki[[74](#_ENREF_74)] | 2013 | India | RHBS | 133 | 3 | 2.26 | 0.47–6.45 |
| Sarkar [[84](#_ENREF_84)] | 2013 | India | PHBS | 900 | 60 | 6.67 | 5.13–8.5 |
| Rizvi [[87](#_ENREF_87)] | 2013 | India | RHBS | 172 | 13 | 7.56 | 4.09–12.58 |
| Pooled |  |  |  | 44478 | 147 | 0.5 | 0.1–0.8 |
