# Supplementary material for: Severe vivax malaria: a systematic review and meta-analysis of clinical studies since 1900
Source: Malar J. 2014 Dec 8;13:481. doi: 10.1186/1475-2875-13-481 (PMC4364574; doi:10.1186/1475-2875-13-481)
Supplement: Supplementary file 8 — Additional file 8: Prevalence of respiratory dysfunction among both outpatients and inpatients of vivax malaria. (DOCX 40 KB) [file 12936_2014_3678_MOESM8_ESM.docx]

**Additional file 8. Prevalence of respiratory dysfunction among both outpatients and inpatients of vivax malaria**

| **Author (Reference)** | **Year** | **Country** | **Study design** | **Total vivax** | **Respiratory dysfunction** | **Prevalence** | **95% CI** |
| --- | --- | --- | --- | --- | --- | --- | --- |
| Svenson[[30](#_ENREF_30)] | 1995 | Canada | RHBS | 246 | 1 | 0.4 | 0.01–2.2 |
| McNeeley[[34](#_ENREF_34)] | 1998 | USA | RHBS | 350 | 1 | 0.3 | 0.01–1.6 |
| Vicas[[39](#_ENREF_39)] | 2005 | USA | RHBS | 30 | 1 | 3.3 | 0.1–17.2 |
| Kochar[[47](#_ENREF_47)] | 2009 | India | PHBS | 456 | 4 | 0.9 | 0.2–2.2 |
| Nayak[[42](#_ENREF_42)] | 2009 | India | PHBS | 169 | 5 | 3.0 | 1.0–7.0 |
| Kochar[[48](#_ENREF_48)] | 2010 | India | PHBS | 103 | 1 | 1.0 | 0.02–5.3 |
| Andrade [[49](#_ENREF_49)] | 2010 | Brazil | PHBS | 129 | 6 | 4.6 | 1.7–9.8 |
| Srivastava [[58](#_ENREF_58)] | 2011 | India | RHBS | 50 | 3 | 6.0 | 1.2–16.5 |
| Mehmood[[68](#_ENREF_68)] | 2012 | Pakistan | RHBS | 97 | 1 | 1.0 | 0.02–5.6 |
| Naha [[15](#_ENREF_15)] | 2012 | India | RHBS | 213 | 4 | 1.9 | 0.5–4.7 |
| Sharma [[69](#_ENREF_69)] | 2012 | India | RHBS | 105 | 9 | 8.6 | 04.0–15.6 |
| Limaye[[16](#_ENREF_16)] | 2012 | India | RHBS | 338 | 10 | 3.0 | 1.4–5.4 |
| Garg [[60](#_ENREF_60)] | 2012 | India | PHBS | 78 | 3 | 3.9 | 0.8–10.8 |
| Mehmood[[68](#_ENREF_68)] | 2012 | Pakistan | RHBS | 97 | 1 | 1 | 0.02–5.6 |
| Naha [[15](#_ENREF_15)] | 2012 | India | RHBS | 213 | 4 | 1.9 | 0.5–4.7 |
| Singh [[73](#_ENREF_73)] | 2013 | India | PHBS | 61 | 5 | 8.2 | 2.71–18.11 |
| Raza [[81](#_ENREF_81)] | 2013 | Pakistan | PHBS | 220 | 31 | 14.09 | 9.78–19.4 |
| Bhatacharjee[[82](#_ENREF_82)] | 2013 | India | RHBS | 168 | 2 | 1.19 | 0.14–4.23 |
| Sarkar [[84](#_ENREF_84)] | 2013 | India | PHBS | 900 | 4 | 0.44 | 0.12–1.13 |
| Aatif[[86](#_ENREF_86)] | 2013 | Pakistan | PHBS | 107 | 1 | 0.93 | 0.02–5.1 |
| Rizvi [[87](#_ENREF_87)] | 2013 | India | RHBS | 172 | 6 | 3.49 | 1.29–7.44 |
| Pooled |  |  |  | 45044 | 98 | 0.1 | 0–0.3 |
