# Supplementary material for: Severe vivax malaria: a systematic review and meta-analysis of clinical studies since 1900
Source: Malar J. 2014 Dec 8;13:481. doi: 10.1186/1475-2875-13-481 (PMC4364574; doi:10.1186/1475-2875-13-481)
Supplement: Supplementary file 9 — Additional file 9: Prevalence of hepatic dysfunction among both outpatients and inpatients of vivax malaria. (DOCX 51 KB) [file 12936_2014_3678_MOESM9_ESM.docx]

**Additional file 9. Prevalence of hepatic dysfunction among both outpatients and inpatients of vivax malaria**

| **Author (Reference)** | **Year** | **Country** | **Study design** | **Total vivax** | **Hepatic dysfunction** | **Prevalence** | **95% CI** |
| --- | --- | --- | --- | --- | --- | --- | --- |
| Read [[22](#_ENREF_22)] | 1946 | USA | PHBS | 211 | 18 | 8.5 | 5.13–13.1 |
| Lippincott [[23](#_ENREF_23)] | 1946 | USA | PHBS | 138 | 3 | 2.2 | 0.4–6.2 |
| Whorton[[24](#_ENREF_24)] | 1947 | USA | PHBS | 320 | 14 | 4.4 | 2.4–7.2 |
| Martelo[[25](#_ENREF_25)] | 1969 | USA | PHBS | 164 | 1 | 0.6 | 0.01–3.3 |
| Singh [[28](#_ENREF_28)] | 1992 | India | PHBS | 25 | 1 | 4.0 | 0.1–20.3 |
| Mohapatra[[37](#_ENREF_37)] | 2002 | India | PHBS | 110 | 8 | 7.2 | 3.2–13.8 |
| Barcus[[12](#_ENREF_12)] | 2007 | Indonesia | RHBS | 1135 | 10 | 0.9 | 0.4–1.6 |
| Beg [[41](#_ENREF_41)] | 2008 | Pakistan | RHBS | 270 | 11 | 4.1 | 2.0–7.2 |
| Sharma [[45](#_ENREF_45)] | 2009 | India | RHBS | 221 | 13 | 5.9 | 3.2–9.8 |
| Kochar[[47](#_ENREF_47)] | 2009 | India | PHBS | 456 | 23 | 5.0 | 3.2–7.5 |
| Khan [[44](#_ENREF_44)] | 2009 | Qatar | PHBS | 39 | 2 | 5.1 | 0.6–17.3 |
| Nayak[[42](#_ENREF_42)] | 2009 | India | PHBS | 169 | 14 | 8.3 | 4.6–13.5 |
| Kochar[[48](#_ENREF_48)] | 2010 | India | PHBS | 103 | 17 | 16.5 | 9.9–25.1 |
| Andrade [[49](#_ENREF_49)] | 2010 | Brazil | PHBS | 129 | 7 | 5.4 | 2.2–10.9 |
| Singh [[59](#_ENREF_59)] | 2011 | India | RHBS | 108 | 4 | 3.7 | 1.0–9.2 |
| Franklin [[53](#_ENREF_53)] | 2011 | Brazil | PHBS | 21 | 1 | 4.8 | 0.1–23.9 |
| Mitja[[54](#_ENREF_54)] | 2011 | PNG | PHBS | 1213 | 6 | 0.5 | 0.2–1.1 |
| Murgod[[56](#_ENREF_56)] | 2011 | India | PHBS | 49 | 9 | 18.4 | 8.8–32.0 |
| Kaushik [[62](#_ENREF_62)] | 2012 | India | PHBS | 35 | 2 | 5.7 | 0.7–19.2 |
| Shaikh [[66](#_ENREF_66)] | 2012 | Pakistan | RHBS | 192 | 1 | 0.5 | 0.01–2.9 |
| Mehmood[[68](#_ENREF_68)] | 2012 | Pakistan | RHBS | 97 | 12 | 12.4 | 6.6–20.6 |
| Naha [[15](#_ENREF_15)] | 2012 | India | RHBS | 213 | 29 | 13.6 | 9.3–19.0 |
| Sharma [[69](#_ENREF_69)] | 2012 | India | RHBS | 105 | 11 | 10.5 | 5.3–18.0 |
| Limaye[[16](#_ENREF_16)] | 2012 | India | RHBS | 338 | 18 | 5.3 | 3.2–8.3 |
| Garg [[60](#_ENREF_60)] | 2012 | India | PHBS | 78 | 23 | 29.5 | 19.7–40.9 |
| Barber [[72](#_ENREF_72)] | 2013 | Malaysia | PHBS | 43 | 2 | 4.6 | 0.6–15.8 |
| Zaki[[74](#_ENREF_74)] | 2013 | India | RHBS | 133 | 1 | 0.75 | 0.02–4.12 |
| Raza [[81](#_ENREF_81)] | 2013 | Pakistan | PHBS | 220 | 26 | 11.82 | 7.87–16.83 |
| Bhatacharjee[[82](#_ENREF_82)] | 2013 | India | RHBS | 168 | 28 | 16.67 | 11.37–23.18 |
| Sarkar [[84](#_ENREF_84)] | 2013 | India | PHBS | 900 | 132 | 14.67 | 12.42–17.15 |
| Rizvi [[87](#_ENREF_87)] | 2013 | India | RHBS | 172 | 18 | 10.46 | 6.32–16.03 |
| Pooled |  |  |  | 45169 | 465 | 2.5 | 1.7–3.4 |
