# Supplementary material for: Severe vivax malaria: a systematic review and meta-analysis of clinical studies since 1900
Source: Malar J. 2014 Dec 8;13:481. doi: 10.1186/1475-2875-13-481 (PMC4364574; doi:10.1186/1475-2875-13-481)
Supplement: Supplementary file 10 — Additional file 10: Prevalence of abnormal bleeding/DIC among both outpatients and inpatients of vivax malaria. (DOCX 39 KB) [file 12936_2014_3678_MOESM10_ESM.docx]

**Additional file 10. Prevalence of abnormal bleeding/DIC among both outpatients and inpatients of vivax malaria**

| **Author (Reference)** | **Year** | **Country** | **Study design** | **Total vivax** | **Abnormal bleeding/DIC** | **Prevalence** | **95% CI** |
| --- | --- | --- | --- | --- | --- | --- | --- |
| Read [[22](#_ENREF_22)] | 1946 | USA | PHBS | 211 | 6 | 2.8 | 1.0–6.1 |
| Whorton[[24](#_ENREF_24)] | 1947 | USA | PHBS | 125 | 3 | 2.4 | 0.5–6.8 |
| Gopinathan[[26](#_ENREF_26)] | 1982 | India | RHBS | 178 | 2 | 1.1 | 0.14–4.0 |
| Kochar[[48](#_ENREF_48)] | 2010 | India | PHBS | 103 | 7 | 6.8 | 2.8–13.5 |
| Singh [[59](#_ENREF_59)] | 2011 | India | RHBS | 108 | 1 | 0.9 | 0.02–5.0 |
| Sharma [[69](#_ENREF_69)] | 2012 | India | RHBS | 105 | 4 | 3.8 | 1.0–9.5 |
| Limaye[[16](#_ENREF_16)] | 2012 | India | RHBS | 338 | 30 | 8.9 | 6.1–12.4 |
| Nurleila[[71](#_ENREF_71)] | 2012 | Indonesia | RHBS | 1837 | 6 | 0.3 | 0.1–0.7 |
| Kaushik [[62](#_ENREF_62)] | 2012 | India | PHBS | 35 | 2 | 5.7 | 0.7–19.2 |
| Tanwar[[64](#_ENREF_64)] | 2012 | India | PHBS | 380 | 63 | 16.6 | 13.0–20.7 |
| Garg [[60](#_ENREF_60)] | 2012 | India | PHBS | 78 | 2 | 2.6 | 0.3–9.0 |
| Barber [[72](#_ENREF_72)] | 2013 | Malaysia | PHBS | 43 | 1 | 2.3 | 0.1–12.3 |
| Zaki[[74](#_ENREF_74)] | 2013 | India | RHBS | 133 | 1 | 0.75 | 0.02–4.12 |
| Bhatacharjee[[82](#_ENREF_82)] | 2013 | India | RHBS | 168 | 9 | 5.36 | 2.48–9.93 |
| Sarkar [[84](#_ENREF_84)] | 2013 | India | PHBS | 900 | 4 | 0.44 | 0.12–1.13 |
| Aatif[[86](#_ENREF_86)] | 2013 | Pakistan | PHBS | 107 | 6 | 5.61 | 2.08–11.81 |
| Rizvi [[87](#_ENREF_87)] | 2013 | India | RHBS | 172 | 8 | 4.65 | 2.029–8.96 |
| Pooled |  |  |  | 44974 | 155 | 0.5 | 0.1–0.8 |
