# Supplementary material for: Severe vivax malaria: a systematic review and meta-analysis of clinical studies since 1900
Source: Malar J. 2014 Dec 8;13:481. doi: 10.1186/1475-2875-13-481 (PMC4364574; doi:10.1186/1475-2875-13-481)
Supplement: Supplementary file 11 — Additional file 11: Prevalence of haemoglobinuria among both outpatients and inpatients of vivax malaria. (DOCX 29 KB) [file 12936_2014_3678_MOESM11_ESM.docx]

**Additional file 11. Prevalence of haemoglobinuria among both outpatients and inpatients of vivax malaria**

| **Author (Reference)** | **Year** | **Country** | **Study design** | **Total vivax** | **Hemoglobinuria** | **Prevalence** | **95% CI** |
| --- | --- | --- | --- | --- | --- | --- | --- |
| Kochar[[48](#_ENREF_48)] | 2010 | India | PHBS | 103 | 2 | 1.9 | 0.2–6.8 |
| Mitja[[54](#_ENREF_54)] | 2011 | PNG | PHBS | 1213 | 1 | 0.1 | 0.002–0.5 |
| Sharma [[69](#_ENREF_69)] | 2012 | India | RHBS | 105 | 5 | 4.8 | 1.6–10.8 |
| Garg [[60](#_ENREF_60)] | 2012 | India | PHBS | 78 | 2 | 2.6 | 0.3–9.0 |
| Singh [[73](#_ENREF_73)] | 2013 | India | PHBS | 61 | 5 | 8.2 | 2.71–18.10 |
| Raza [[81](#_ENREF_81)] | 2013 | Pakistan | PHBS | 220 | 5 | 2.27 | 0.74–5.22 |
| Sarkar [[84](#_ENREF_84)] | 2013 | India | PHBS | 900 | 8 | 0.89 | 0.38–1.74 |
| Pooled |  |  |  | 45044 | 28 | 1.4 | 0.4–2.4 |
