# Supplementary material for: Severe vivax malaria: a systematic review and meta-analysis of clinical studies since 1900
Source: Malar J. 2014 Dec 8;13:481. doi: 10.1186/1475-2875-13-481 (PMC4364574; doi:10.1186/1475-2875-13-481)
Supplement: Supplementary file 12 — Additional file 12: Prevalence of hypoglycaemia among both outpatients and inpatients of vivax malaria. (DOCX 30 KB) [file 12936_2014_3678_MOESM12_ESM.docx]

**Additional file 12. Prevalence of hypoglycemia among both outpatients and inpatients of vivax malaria**

| **Author (Reference)** | **Year** | **Country** | **Study design** | **Total vivax** | **Hypoglycemia** | **Prevalence** | **95% CI** |
| --- | --- | --- | --- | --- | --- | --- | --- |
| Kochar[[47](#_ENREF_47)] | 2009 | India | PHBS | 456 | 1 | 0.2 | 0.006–1.2 |
| Nayak[[42](#_ENREF_42)] | 2009 | India | PHBS | 169 | 5 | 3.0 | 1.0–6.8 |
| Shaikh [[66](#_ENREF_66)] | 2012 | Pakistan | RHBS | 192 | 5 | 2.6 | 0.8–6.0 |
| Sharma [[69](#_ENREF_69)] | 2012 | India | RHBS | 105 | 3 | 2.9 | 0.6–8.1 |
| Singh [[73](#_ENREF_73)] | 2013 | India | PHBS | 61 | 4 | 6.56 | 1.81–15.95 |
| Bhatacharjee[[82](#_ENREF_82)] | 2013 | India | RHBS | 168 | 5 | 2.98 | 0.97–6.81 |
| Ketema[[83](#_ENREF_83)] | 2013 | Ethiopia | PHBS | 139 | 2 | 1.44 | 0.17–5.1 |
| Rizvi [[87](#_ENREF_87)] | 2013 | India | RHBS | 172 | 3 | 1.74 | 0.36–5.01 |
| Pooled |  |  |  | 45014 | 28 | 2.0 | 0.8–3.2 |
