# Supplementary material for: Severe vivax malaria: a systematic review and meta-analysis of clinical studies since 1900
Source: Malar J. 2014 Dec 8;13:481. doi: 10.1186/1475-2875-13-481 (PMC4364574; doi:10.1186/1475-2875-13-481)
Supplement: Supplementary file 13 — Additional file 13: Prevalence of metabolic acidosis among both outpatients and inpatients of vivax malaria. (DOCX 28 KB) [file 12936_2014_3678_MOESM13_ESM.docx]

**Additional file 13. Prevalence of metabolic acidosis among both outpatients and inpatients of vivax malaria**

| **Author (Reference)** | **Year** | **Country** | **Study design** | **Total vivax** | **Metabolic acidosis** | **Prevalence** | **95% CI** |
| --- | --- | --- | --- | --- | --- | --- | --- |
| Barcus[[12](#_ENREF_12)] | 2007 | Indonesia | RHBS | 1135 | 3 | 0.3 | 0.05–0.8 |
| Kochar[[48](#_ENREF_48)] | 2010 | India | PHBS | 103 | 1 | 1.0 | 0.02–5.3 |
| Limaye[[16](#_ENREF_16)] | 2012 | India | RHBS | 338 | 14 | 4.1 | 2.3–6.8 |
| Raza [[81](#_ENREF_81)] | 2013 | Pakistan | PHBS | 220 | 20 | 9.09 | 5.64–13.69 |
| Pooled |  |  |  | 44478 | 38 | 0.4 | 0.1–0.7 |
