# Supplementary material for: Severe vivax malaria: a systematic review and meta-analysis of clinical studies since 1900
Source: Malar J. 2014 Dec 8;13:481. doi: 10.1186/1475-2875-13-481 (PMC4364574; doi:10.1186/1475-2875-13-481)
Supplement: Supplementary file 14 — Additional file 14: Prevalence of shock among both outpatients and inpatients of vivax malaria. (DOCX 29 KB) [file 12936_2014_3678_MOESM14_ESM.docx]

**Additional file 14. Prevalence of shock among both outpatients and inpatients of vivax malaria**

| **Author (Reference)** | **Year** | **Country** | **Study design** | **Total vivax** | **Shock** | **Prevalence** | **95% CI** |
| --- | --- | --- | --- | --- | --- | --- | --- |
| Garg [[60](#_ENREF_60)] | 2012 | India | PHBS | 78 | 1 | 1.28 | 0.03–7.0 |
| Limaye[[16](#_ENREF_16)] | 2012 | India | RHBS | 338 | 18 | 5.3 | 3.2–8.3 |
| Barber [[72](#_ENREF_72)] | 2013 | Malaysia | PHBS | 43 | 1 | 2.3 | 0.1–12.3 |
| Singh [[73](#_ENREF_73)] | 2013 | India | PHBS | 61 | 4 | 6.56 | 1.81–15.95 |
| Zaki[[74](#_ENREF_74)] | 2013 | India | RHBS | 133 | 1 | 0.75 | 0.02–4.12 |
| Rizvi [[87](#_ENREF_87)] | 2013 | India | RHBS | 172 | 10 | 5.814 | 2.82–10.43 |
| Pooled |  |  |  | 45044 | 35 | 3.3 | 1.1–5.4 |
