# Supplementary material for: Severe vivax malaria: a systematic review and meta-analysis of clinical studies since 1900
Source: Malar J. 2014 Dec 8;13:481. doi: 10.1186/1475-2875-13-481 (PMC4364574; doi:10.1186/1475-2875-13-481)
Supplement: Supplementary file 15 — Additional file 15: Prevalence of severe anaemia among both outpatients and inpatients of vivax malaria. (DOCX 219 KB) [file 12936_2014_3678_MOESM15_ESM.docx]

**Additional file 15. Prevalence of severe anemia among both outpatients and inpatients of vivax malaria**

| **Author (Reference)** | **Year** | **Country** | **Study design** | **Total vivax** | **Severe anemia** | **Prevalence** | **95% CI** |
| --- | --- | --- | --- | --- | --- | --- | --- |
| Lynk[[27](#_ENREF_27)] | 1989 | Canada | RHBS | 24 | 1 | 4.2 | 0.1–21.1 |
| Rodriguez-Morales [[40](#_ENREF_40)] | 2006 | Venezuela | PHBS | 78 | 8 | 10.2 | 4.5–19.2 |
| Genton[[14](#_ENREF_14)] | 2008 | PNG | PHBS | 1946 | 16 | 0.8 | 0.5–1.3 |
| Tjitra[[13](#_ENREF_13)] | 2008 | Indonesia | PHBS | 2937 | 589 | 20.0 | 18.6–21.5 |
| Poespoprodjo[[43](#_ENREF_43)] | 2009 | Indonesia | PHBS | 668 | 193 | 28.9 | 25.5–32.5 |
| Kochar[[47](#_ENREF_47)] | 2009 | India | PHBS | 456 | 13 | 2.8 | 1.5–4.8 |
| Nayak[[42](#_ENREF_42)] | 2009 | India | PHBS | 169 | 55 | 32.5 | 25.5–40.2 |
| Kochar[[48](#_ENREF_48)] | 2010 | India | PHBS | 103 | 49 | 47.6 | 37.6–57.6 |
| Singh [[59](#_ENREF_59)] | 2011 | India | RHBS | 108 | 8 | 7.4 | 3.2–14.1 |
| Mitja[[54](#_ENREF_54)] | 2011 | PNG | PHBS | 1213 | 3 | 0.2 | 0.05–0.7 |
| Deepa[[55](#_ENREF_55)] | 2011 | India | PHBS | 37 | 1 | 2.7 | 0.1–14.2 |
| Kaushik [[62](#_ENREF_62)] | 2012 | India | PHBS | 35 | 7 | 20.0 | 8.4–36.9 |
| Shaikh [[66](#_ENREF_66)] | 2012 | Pakistan | RHBS | 192 | 11 | 5.7 | 2.9–10.0 |
| Naha [[15](#_ENREF_15)] | 2012 | India | RHBS | 213 | 1 | 0.5 | 0.01–2.6 |
| Sharma [[69](#_ENREF_69)] | 2012 | India | RHBS | 105 | 19 | 18.1 | 11.3–26.9 |
| Limaye[[16](#_ENREF_16)] | 2012 | India | RHBS | 338 | 10 | 3.0 | 1.4–5.4 |
| Garg [[60](#_ENREF_60)] | 2012 | India | PHBS | 78 | 2 | 2.6 | 0.3–9.0 |
| Singh [[73](#_ENREF_73)] | 2013 | India | PHBS | 61 | 6 | 9.84 | 3.7–20.19 |
| Douglas [[75](#_ENREF_75)] | 2013 | Indonesia | RHBS | 19858 | 1050 | 5.29 | 4.98–5.61 |
| Bhatacharjee[[82](#_ENREF_82)] | 2013 | India | RHBS | 168 | 14 | 8.33 | 4.63–13.59 |
| Ketema[[83](#_ENREF_83)] | 2013 | Ethiopia | PHBS | 139 | 8 | 5.75 | 2.52–11.02 |
| Sarkar [[84](#_ENREF_84)] | 2013 | India | PHBS | 900 | 12 | 1.33 | 0.69–2.32 |
| Rizvi [[87](#_ENREF_87)] | 2013 | India | RHBS | 172 | 16 | 9.30 | 5.41–14.67 |
| Jain [[89](#_ENREF_89)] | 2013 | India | PHBS | 198 | 7 | 3.53 | 1.43–7.15 |
| Pooled |  |  |  | 45044 | 2099 | 2.8 | 1.8–3.9 |

**Additional file 16. Prevalence of severe thrombocytopenia among both outpatients and inpatients of vivax malaria**

| **Author (Reference)** | **Year** | **Country** | **Study design** | **Total vivax** | **Severe thrombocytopenia** | **Prevalence** | **95% CI** |
| --- | --- | --- | --- | --- | --- | --- | --- |
| Lynk[[27](#_ENREF_27)] | 1989 | Canada | RHBS | 24 | 2 | 8.33 | 1.03–27.0 |
| Lee [[32](#_ENREF_32)] | 1997 | Thailand | PHBS | 24 | 3 | 12.5 | 2.7–32.4 |
| Oh [[35](#_ENREF_35)] | 2001 | S. Korea | RHBS | 101 | 5 | 4.9 | 1.6–11.2 |
| Song [[38](#_ENREF_38)] | 2003 | S. Korea | RHBS | 44 | 9 | 20.4 | 9.8–35.3 |
| Sharma [[45](#_ENREF_45)] | 2009 | India | RHBS | 221 | 13 | 5.9 | 3.2–9.8 |
| Poespoprodjo[[43](#_ENREF_43)] | 2009 | Indonesia | PHBS | 102 | 23 | 22.5 | 14.9–31.9 |
| Singh [[59](#_ENREF_59)] | 2011 | India | RHBS | 108 | 9 | 8.3 | 3.9–15.2 |
| Mitja[[54](#_ENREF_54)] | 2011 | PNG | PHBS | 1213 | 3 | 0.2 | 0.05–0.7 |
| Shaikh [[66](#_ENREF_66)] | 2012 | Pakistan | RHBS | 192 | 19 | 9.9 | 6.1–15.0 |
| Mehmood[[68](#_ENREF_68)] | 2012 | Pakistan | RHBS | 97 | 44 | 45.4 | 35.2–55.8 |
| Naha [[15](#_ENREF_15)] | 2012 | India | RHBS | 213 | 68 | 32.0 | 26.0–39.0 |
| Sharma [[69](#_ENREF_69)] | 2012 | India | RHBS | 105 | 62 | 59.0 | 49.0–68.5 |
| Tanwar[[64](#_ENREF_64)] | 2012 | India | PHBS | 380 | 144 | 37.9 | 33.0–43.0 |
| Garg [[60](#_ENREF_60)] | 2012 | India | PHBS | 78 | 2 | 2.6 | 0.3–9.0 |
| Barber [[72](#_ENREF_72)] | 2013 | Malaysia | PHBS | 43 | 19 | 44.2 | 29.1–60.1 |
| Singh [[73](#_ENREF_73)] | 2013 | India | PHBS | 61 | 18 | 29.51 | 18.52–42.57 |
| Leal-Santos [[80](#_ENREF_80)] | 2013 | Brazil | PHBS | 186 | 31 | 16.66 | 11.61–22.81 |
| Bhatacharjee[[82](#_ENREF_82)] | 2013 | India | RHBS | 168 | 12 | 7.14 | 3.74–12.14 |
| Sarkar [[84](#_ENREF_84)] | 2013 | India | PHBS | 900 | 24 | 2.67 | 1.72–3.94 |
| Aatif[[86](#_ENREF_86)] | 2013 | Pakistan | PHBS | 107 | 96 | 89.72 | 82.34–94.75 |
| Kwak[[88](#_ENREF_88)] | 2013 | South Korea | RHBS | 352 | 148 | 42.04 | 36.83–47.39 |
| Pooled |  |  |  | 44478 | 754 | 7.5 | 4.2–10.8 |

**Additional file 17. Prevalence of death among both outpatients and inpatients of vivax malaria**

| **Author (Reference)** | **Year** | **Country** | **Study design** | **Total vivax** | **Death** | **Prevalence** | **95% CI** |
| --- | --- | --- | --- | --- | --- | --- | --- |
| Bahr [[18](#_ENREF_18)] | 1928 | USA | PHBS | 100 | 3 | 3.0 | 0.6–8.5 |
| Fitz-Hugh [[20](#_ENREF_20)] | 1944 | India | RHBS | 1375 | 1 | 0.1 | 0.002–0.4 |
| Whorton[[24](#_ENREF_24)] | 1947 | USA | PHBS | 195 | 1 | 0.5 | 0.01–2.8 |
| Singh [[28](#_ENREF_28)] | 1992 | India | PHBS | 25 | 1 | 4.0 | 0.1–20.3 |
| Wattanagoon[[29](#_ENREF_29)] | 1994 | Thailand | RHBS | 460 | 1 | 0.22 | 0.005–1.2 |
| Svenson[[30](#_ENREF_30)] | 1995 | Canada | RHBS | 246 | 1 | 0.4 | 0.01–2.2 |
| Vicas[[39](#_ENREF_39)] | 2005 | USA | RHBS | 30 | 1 | 3.3 | 0.1–17.2 |
| Barcus[[12](#_ENREF_12)] | 2007 | Indonesia | RHBS | 1135 | 9 | 0.8 | 0.4–1.5 |
| Beg [[41](#_ENREF_41)] | 2008 | Pakistan | RHBS | 270 | 4 | 1.5 | 0.4–3.7 |
| Tjitra[[13](#_ENREF_13)] | 2008 | Indonesia | PHBS | 2937 | 46 | 1.6 | 1.1–2.1 |
| Poespoprodjo[[43](#_ENREF_43)] | 2009 | Indonesia | PHBS | 668 | 6 | 0.9 | 0.3–1.9 |
| Kochar[[47](#_ENREF_47)] | 2009 | India | PHBS | 456 | 2 | 0.4 | 0.05–1.6 |
| Sharma [[45](#_ENREF_45)] | 2009 | India | RHBS | 221 | 3 | 1.4 | 0.3–3.9 |
| Kochar[[48](#_ENREF_48)] | 2010 | India | PHBS | 103 | 4 | 3.9 | 1.1–9.6 |
| Andrade [[49](#_ENREF_49)] | 2010 | Brazil | PHBS | 129 | 6 | 4.6 | 1.7–9.8 |
| Singh [[59](#_ENREF_59)] | 2011 | India | RHBS | 108 | 1 | 0.9 | 0.02–5.0 |
| Shaikh [[66](#_ENREF_66)] | 2012 | Pakistan | RHBS | 192 | 1 | 0.5 | 0.01–2.9 |
| Sharma [[69](#_ENREF_69)] | 2012 | India | RHBS | 105 | 10 | 9.5 | 4.7–16.8 |
| Limaye[[16](#_ENREF_16)] | 2012 | India | RHBS | 338 | 6 | 1.8 | 0.6–3.8 |
| Nurleila[[71](#_ENREF_71)] | 2012 | Indonesia | RHBS | 1837 | 18 | 1.0 | 0.6–1.5 |
| Garg [[60](#_ENREF_60)] | 2012 | India | PHBS | 78 | 1 | 1.3 | 0.03–6.9 |
| Singh [[73](#_ENREF_73)] | 2013 | India | PHBS | 61 | 5 | 8.2 | 2.7–18.1 |
| Zaki[[74](#_ENREF_74)] | 2013 | India | RHBS | 133 | 1 | 0.75 | 0.02–4.12 |
| Douglas [[75](#_ENREF_75)] | 2013 | Indonesia | RHBS | 19858 | 112 | 0.6 | 0.5–0.7 |
| Sarkar [[84](#_ENREF_84)] | 2013 | India | PHBS | 900 | 40 | 4.44 | 3.19–6.0 |
| Aatif[[86](#_ENREF_86)] | 2013 | Pakistan | PHBS | 107 | 1 | 0.93 | 0.02–5.1 |
| Rizvi [[87](#_ENREF_87)] | 2013 | India | RHBS | 172 | 3 | 1.74 | 0.36–5.01 |
| Jain [[89](#_ENREF_89)] | 2013 | India | PHBS | 198 | 2 | 1.01 | 0.122–3.6 |
| Pooled |  |  |  | 45044 | 290 | 0.2 | 0.1–0.3 |

**Additional file 18 Pooled prevalence of severity signs among both inpatients and outpatients of vivax malaria (62 studies)**

| Complication | Total vivax | Total patients with severity sign | Pooled prevalence, % | 95% CI, % |
| --- | --- | --- | --- | --- |
| Death | 45044 | 290 | 0.2 | 0.1–0.3 |
| Cerebral malaria | 44478 | 410 | 0.6 | 0.3–0.8 |
| Multiple convulsions | 45014 | 70 | 0.1 | 0–0.3 |
| Renal dysfunction | 44478 | 147 | 0.5 | 0.1–0.8 |
| Respiratory dysfunction | 45044 | 98 | 0.1 | 0–0.3 |
| Hepatic dysfunction | 45169 | 465 | 2.5 | 1.7–3.4 |
| Abnormal bleeding/DIC | 44974 | 155 | 0.5 | 0.1–0.8 |
| Haemoglobinuria | 45044 | 28 | 1.4 | 0.4–2.4 |
| Hypoglycaemia | 45014 | 28 | 2 | 0.8–3.2 |
| Metabolic acidosis | 44478 | 38 | 0.4 | 0.1–0.7 |
| Circulatory collapse/Shock | 45044 | 35 | 3.3 | 1.1–5.4 |
| Severe anaemia | 45044 | 2099 | 2.8 | 1.8–3.9 |
| Severe thrombocytopaenia | 44478 | 754 | 7.5 | 4.2–10.8 |

**Additional file 19. Prevalence of cerebral malaria among only inpatients of vivax malaria**

| Author (Reference) | Year | Country | Study design | Total vivax | Cerebral malaria | Prevalence | 95% CI |
| --- | --- | --- | --- | --- | --- | --- | --- |
| George [[50](#_ENREF_50)] | 2010 | India | RHBS | 30 | 2 | 6.7 | 0.8–22.1 |
| Manning [[51](#_ENREF_51)] | 2011 | PNG | PHBS | 27 | 7 | 25.9 | 11.1–46.3 |
| Yadav [[65](#_ENREF_65)] | 2012 | India | RHBS | 131 | 22 | 16.8 | 10.8–24.3 |
| Mahgoub[[61](#_ENREF_61)] | 2012 | Sudan | PHBS | 18 | 2 | 11.1 | 1.4–34.7 |
| Nadkar[[63](#_ENREF_63)] | 2012 | India | PHBS | 488 | 40 | 8.2 | 5.9–11 |
| Lanca[[67](#_ENREF_67)] | 2012 | Brazil | RHBS | 24 | 5 | 20.8 | 7.1–42.1 |
| Lon [[76](#_ENREF_76)] | 2013 | Cambodia | RHBS | 33 | 16 | 48.5 | 30.8–66.4 |
| Abdallah [[77](#_ENREF_77)] | 2013 | Sudan | PHBS | 26 | 3 | 11.54 | 2.45–30.15 |
| Sharma [[78](#_ENREF_78)] | 2013 | India | RHBS | 54 | 10 | 18.52 | 9.2–31.43 |
| Gehlawat[[79](#_ENREF_79)] | 2013 | India | PHBS | 18 | 9 | 50 | 26.02–73.98 |
| Zubairi[[85](#_ENREF_85)] | 2013 | Pakistan | RHBS | 296 | 6 | 2.03 | 0.75–4.36 |
| Pooled |  |  |  | 1367 | 122 | 10.1 | 5.6–14.7 |

**Additional file 20. Prevalence of repeated generalized seizures among only inpatients of vivax malaria**

| Author (Reference) | Year | Country | Study design | Total vivax | Repeated generalized seizures | Prevalence | 95% CI |
| --- | --- | --- | --- | --- | --- | --- | --- |
| Mahgoub[[61](#_ENREF_61)] | 2012 | Sudan | PHBS | 18 | 3 | 16.7 | 3.6–41.4 |
| Lon [[76](#_ENREF_76)] | 2013 | Cambodia | RHBS | 33 | 2 | 6.06 | 0.74–20.23 |
| Abdallah [[77](#_ENREF_77)] | 2013 | Sudan | PHBS | 26 | 1 | 3.85 | 0.1–19.637 |
| Sharma [[78](#_ENREF_78)] | 2013 | India | RHBS | 54 | 4 | 7.41 | 2.05–17.89 |
| Gehlawat[[79](#_ENREF_79)] | 2013 | India | PHBS | 18 | 8 | 44.44 | 21.53–69.24 |
| Pooled |  |  |  | 1372 | 18 | 13.7 | 11.5–15.8 |

**Additional file 21. Prevalence of renal dysfunction among only inpatients of vivax malaria**

| **Author (Reference)** | **Year** | **Country** | **Study design** | **Total vivax** | **Renal dysfunction** | **Prevalence** | **95% CI** |
| --- | --- | --- | --- | --- | --- | --- | --- |
| George [[50](#_ENREF_50)] | 2010 | India | RHBS | 30 | 1 | 3.3 | 0.1–17.2 |
| Manning [[51](#_ENREF_51)] | 2011 | PNG | PHBS | 27 | 1 | 3.7 | 0.1–19.0 |
| Yadav [[65](#_ENREF_65)] | 2012 | India | RHBS | 131 | 8 | 6.1 | 2.7–11.7 |
| Lanca[[67](#_ENREF_67)] | 2012 | Brazil | RHBS | 24 | 2 | 8.3 | 1.0–27.0 |
| Nandwani[[70](#_ENREF_70)] | 2012 | India | RHBS | 110 | 75 | 68.2 | 58.6–76.7 |
| Zubairi[[85](#_ENREF_85)] | 2013 | Pakistan | RHBS | 296 | 10 | 3.38 | 1.63–6.12 |
| Pooled |  |  |  | 1367 | 97 | 5.9 | 0–14.1 |

**Additional file 22. Prevalence of respiratory dysfunction among only inpatients of vivax malaria**

| **Author (Reference)** | **Year** | **Country** | **Study design** | **Total vivax** | **Respiratory dysfunction** | **Prevalence** | **95% CI** |
| --- | --- | --- | --- | --- | --- | --- | --- |
| George [[50](#_ENREF_50)] | 2010 | India | RHBS | 30 | 1 | 3.3 | 0.1–17.2 |
| Nayak[[52](#_ENREF_52)] | 2011 | India | PHBS | 80 | 4 | 5.0 | 1.4–12.3 |
| Nadkar[[63](#_ENREF_63)] | 2012 | India | PHBS | 488 | 8 | 1.6 | 0.7–3.2 |
| Yadav [[65](#_ENREF_65)] | 2012 | India | RHBS | 131 | 3 | 2.3 | 0.5–6.5 |
| Lanca[[67](#_ENREF_67)] | 2012 | Brazil | RHBS | 24 | 3 | 6.7 | 2.7–32.4 |
| Lon [[76](#_ENREF_76)] | 2013 | Cambodia | RHBS | 33 | 1 | 3.03 | 0.08–15.76 |
| Sharma [[78](#_ENREF_78)] | 2013 | India | RHBS | 54 | 3 | 5.55 | 1.16–15.39 |
| Zubairi[[85](#_ENREF_85)] | 2013 | Pakistan | RHBS | 296 | 23 | 7.77 | 4.99–11.43 |
| Pooled |  |  |  | 1367 | 46 | 1.0 | 0.1–1.8 |

**Additional file 23. Prevalence of hepatic dysfunction among only inpatients of vivax malaria**

| **Author (Reference)** | **Year** | **Country** | **Study design** | **Total vivax** | **Hepatic dysfunction** | **Prevalence** | **95% CI** |
| --- | --- | --- | --- | --- | --- | --- | --- |
| Rodriguez-Morales [[46](#_ENREF_46)] | 2009 | Venezuela | RHBS | 17 | 6 | 35.3 | 14.2–61.7 |
| George [[50](#_ENREF_50)] | 2010 | India | RHBS | 30 | 13 | 43.3 | 25.5–62.6 |
| Nayak[[52](#_ENREF_52)] | 2011 | India | PHBS | 80 | 23 | 28.75 | 19.18–39.95 |
| Mahgoub[[61](#_ENREF_61)] | 2012 | Sudan | PHBS | 18 | 5 | 27.8 | 9.7–53.5 |
| Nadkar[[63](#_ENREF_63)] | 2012 | India | PHBS | 488 | 95 | 19.5 | 16.0–23.3 |
| Lanca[[67](#_ENREF_67)] | 2012 | Brazil | RHBS | 24 | 6 | 8.9 | 9.8–46.7 |
| Nandwani[[70](#_ENREF_70)] | 2012 | India | RHBS | 110 | 60 | 54.5 | 44.8–64.1 |
| Lon [[76](#_ENREF_76)] | 2013 | Cambodia | RHBS | 33 | 4 | 12.12 | 3.40–28.2 |
| Abdallah [[77](#_ENREF_77)] | 2013 | Sudan | PHBS | 26 | 3 | 11.54 | 2.45–30.15 |
| Sharma [[78](#_ENREF_78)] | 2013 | India | RHBS | 54 | 14 | 25.92 | 14.96–39.65 |
| Gehlawat[[79](#_ENREF_79)] | 2013 | India | PHBS | 18 | 5 | 27.78 | 9.69–53.5 |
| Zubairi[[85](#_ENREF_85)] | 2013 | Pakistan | RHBS | 296 | 28 | 9.46 | 6.38–13.38 |
| Pooled |  |  |  | 1367 | 262 | 19.5 | 12.6–26.3 |

**Additional file 24. Prevalence of abnormal bleeding/DIC among only inpatients of vivax malaria**

| **Author (Reference)** | **Year** | **Country** | **Study design** | **Total vivax** | **Abnormal bleeding/DIC** | **Prevalence** | **95% CI** |
| --- | --- | --- | --- | --- | --- | --- | --- |
| Rodriguez-Morales [[46](#_ENREF_46)] | 2009 | Venezuela | RHBS | 17 | 4 | 23.5 | 6.8–49.9 |
| Yadav [[65](#_ENREF_65)] | 2012 | India | RHBS | 131 | 12 | 9.1 | 4.8–15.4 |
| Mahgoub[[61](#_ENREF_61)] | 2012 | Sudan | PHBS | 18 | 2 | 11.1 | 1.4–34.7 |
| Lon [[76](#_ENREF_76)] | 2013 | Cambodia | RHBS | 33 | 5 | 15.15 | 5.11–31.9 |
| Abdallah [[77](#_ENREF_77)] | 2013 | Sudan | PHBS | 26 | 2 | 7.69 | 0.94–25.13 |
| Sharma [[78](#_ENREF_78)] | 2013 | India | RHBS | 54 | 10 | 18.52 | 9.25–31.43 |
| Zubairi[[85](#_ENREF_85)] | 2013 | Pakistan | RHBS | 296 | 16 | 5.4 | 3.12–8.63 |
| Pooled |  |  |  | 1372 | 51 | 10.1 | 5.9–14.3 |

**Additional file 25. Prevalence of haemoglobinuria among only inpatients of vivax malaria**

| **Author (Reference)** | **Year** | **Country** | **Study design** | **Total vivax** | **Hemoglobinuria** | **Prevalence** | **95% CI** |
| --- | --- | --- | --- | --- | --- | --- | --- |
| Lanca[[67](#_ENREF_67)] | 2012 | Brazil | RHBS | 24 | 2 | 8.3 | 1.0–27.0 |
| Sharma [[78](#_ENREF_78)] | 2013 | India | RHBS | 54 | 1 | 1.85 | 0.047–9.89 |
| Zubairi[[85](#_ENREF_85)] | 2013 | Pakistan | RHBS | 296 | 62 | 20.95 | 16.45–26.03 |
| Pooled |  |  |  | 1367 | 65 | 1.9 | 0–7.0 |

**Additional file 26. Prevalence of hypoglycaemia among only inpatients of vivax malaria**

| **Author (Reference)** | **Year** | **Country** | **Study design** | **Total vivax** | **Hypoglycemia** | **Prevalence** | **95% CI** |
| --- | --- | --- | --- | --- | --- | --- | --- |
| Mahgoub[[61](#_ENREF_61)] | 2012 | Sudan | PHBS | 18 | 5 | 27.8 | 9.7–53.5 |
| Yadav [[65](#_ENREF_65)] | 2012 | India | RHBS | 131 | 1 | 0.7 | 0.02–4.2 |
| Lanca[[67](#_ENREF_67)] | 2012 | Brazil | RHBS | 24 | 3 | 12.5 | 2.7–32.4 |
| Nandwani[[70](#_ENREF_70)] | 2012 | India | RHBS | 110 | 2 | 1.8 | 0.2–6.4 |
| Abdallah [[77](#_ENREF_77)] | 2013 | Sudan | PHBS | 26 | 2 | 5.23 | 0.94–25.13 |
| Zubairi[[85](#_ENREF_85)] | 2013 | Pakistan | RHBS | 296 | 3 | 1.01 | 0.21–2.93 |
| Pooled |  |  |  | 1367 | 16 | 1.7 | 0–3.6 |

**Additional file 27. Prevalence of metabolic acidosis among only inpatients of vivax malaria**

| **Author (Reference)** | **Year** | **Country** | **Study design** | **Total vivax** | **Metabolic acidosis** | **Prevalence** | **95% CI** |
| --- | --- | --- | --- | --- | --- | --- | --- |
| Manning [[51](#_ENREF_51)] | 2011 | PNG | PHBS | 27 | 3 | 11.1 | 2.3–29.2 |
| Lanca[[67](#_ENREF_67)] | 2012 | Brazil | RHBS | 24 | 10 | 41.7 | 22.1–63.4 |
| Nandwani[[70](#_ENREF_70)] | 2012 | India | RHBS | 110 | 70 | 63.6 | 53.9–72.6 |
| Zubairi[[85](#_ENREF_85)] | 2013 | Pakistan | RHBS | 296 | 17 | 5.74 | 3.38–9.04 |
| Pooled |  |  |  | 1367 | 100 | 7.8 | 0–23.3 |

**Additional file 28. Prevalence of shock among only inpatients of vivax malaria**

| **Author (Reference)** | **Year** | **Country** | **Study design** | **Total vivax** | **Shock** | **Prevalence** | **95% CI** |
| --- | --- | --- | --- | --- | --- | --- | --- |
| Lanca[[67](#_ENREF_67)] | 2012 | Brazil | RHBS | 24 | 13 | 54.2 | 32.9–74.4 |
| Lon [[76](#_ENREF_76)] | 2013 | Cambodia | RHBS | 33 | 7 | 21.21 | 8.98–38.91 |
| Abdallah [[77](#_ENREF_77)] | 2013 | Sudan | PHBS | 26 | 4 | 15.38 | 4.36–34.87 |
| Gehlawat[[79](#_ENREF_79)] | 2013 | India | PHBS | 18 | 3 | 16.67 | 3.58–41.42 |
| Zubairi[[85](#_ENREF_85)] | 2013 | Pakistan | RHBS | 296 | 5 | 1.69 | 0.55–3.9 |
| Pooled |  |  |  | 1367 | 32 | 5.7 | 0–13.3 |

**Additional file 29. Prevalence of severe anaemia among only inpatients of vivax malaria**

| **Author (Reference)** | **Year** | **Country** | **Study design** | **Total vivax** | **Severe anemia** | **Prevalence** | **95% CI** |
| --- | --- | --- | --- | --- | --- | --- | --- |
| George [[50](#_ENREF_50)] | 2010 | India | RHBS | 30 | 3 | 10.0 | 2.1–26.5 |
| Manning [[51](#_ENREF_51)] | 2011 | PNG | PHBS | 27 | 4 | 14.9 | 4.2–33.7 |
| Nayak[[52](#_ENREF_52)] | 2011 | India | PHBS | 80 | 26 | 32.5 | 22.4–43.9 |
| Mahgoub[[61](#_ENREF_61)] | 2012 | Sudan | PHBS | 18 | 6 | 33.3 | 13.3–59.0 |
| Yadav [[65](#_ENREF_65)] | 2012 | India | RHBS | 131 | 45 | 34.3 | 26.3–43.1 |
| Lanca[[67](#_ENREF_67)] | 2012 | Brazil | RHBS | 24 | 4 | 16.7 | 4.7–37.4 |
| Nandwani[[70](#_ENREF_70)] | 2012 | India | RHBS | 110 | 65 | 59.1 | 49.3–68.4 |
| Lon [[76](#_ENREF_76)] | 2013 | Cambodia | RHBS | 33 | 11 | 33.33 | 17.96–51.83 |
| Sharma [[78](#_ENREF_78)] | 2013 | India | RHBS | 54 | 8 | 14.81 | 6.62–27.12 |
| Gehlawat[[79](#_ENREF_79)] | 2013 | India | PHBS | 18 | 5 | 27.78 | 9.69–53.48 |
| Pooled |  |  |  | 1367 | 177 | 17.3 | 9.1–25.4 |

**Additional file 30. Prevalence of severe thrombocytopaenia among only inpatients of vivax malaria**

| **Author (Reference)** | **Year** | **Country** | **Study design** | **Total vivax** | **Severe thrombocytopenia** | **Prevalence** | **95% CI** |
| --- | --- | --- | --- | --- | --- | --- | --- |
| George [[50](#_ENREF_50)] | 2010 | India | RHBS | 30 | 28 | 93.3 | 77.9–99.2 |
| Yadav [[65](#_ENREF_65)] | 2012 | India | RHBS | 131 | 17 | 13.0 | 7.7–20.0 |
| Lanca[[67](#_ENREF_67)] | 2012 | Brazil | RHBS | 24 | 7 | 29.2 | 12.6–51.1 |
| Mahgoub[[61](#_ENREF_61)] | 2012 | Sudan | PHBS | 18 | 4 | 22.2 | 6.4–47.6 |
| Sharma [[78](#_ENREF_78)] | 2013 | India | RHBS | 54 | 20 | 37.04 | 24.29–51.26 |
| Zubairi[[85](#_ENREF_85)] | 2013 | Pakistan | RHBS | 296 | 58 | 19.59 | 15.23–24.58 |
| Pooled |  |  |  | 1367 | 134 | 13.9 | 0–29.1 |

**Additional file 31. Prevalence of death among only inpatients of vivax malaria**

| **Author (Reference)** | **Year** | **Country** | **Study design** | **Total vivax** | **Death** | **Prevalence** | **95% CI** |
| --- | --- | --- | --- | --- | --- | --- | --- |
| Giglioli[[19](#_ENREF_19)] | 1930 | British Guiana | RHBS | 15 | 3 | 20.0 | 4.3–48.1 |
| Rodriguez-Morales  [[46](#_ENREF_46)] | 2009 | Venezuela | RHBS | 17 | 1 | 5.9 | 0.1–28.7 |
| George [[50](#_ENREF_50)] | 2010 | India | RHBS | 30 | 2 | 6.7 | 0.8–22.1 |
| Manning [[51](#_ENREF_51)] | 2011 | PNG | PHBS | 27 | 1 | 3.7 | 0.1–19.0 |
| Nadkar[[63](#_ENREF_63)] | 2012 | India | PHBS | 488 | 44 | 9.0 | 6.6–11.9 |
| Yadav [[65](#_ENREF_65)] | 2012 | India | RHBS | 131 | 4 | 3.0 | 0.8–7.6 |
| Lanca[[67](#_ENREF_67)] | 2012 | Brazil | RHBS | 24 | 2 | 8.3 | 1.0–27.0 |
| Lon [[76](#_ENREF_76)] | 2013 | Cambodia | RHBS | 33 | 4 | 12.12 | 3.4–28.2 |
| Gehlawat[[79](#_ENREF_79)] | 2013 | India | PHBS | 18 | 2 | 11.11 | 1.37–34.7 |
| Pooled |  |  |  | 1367 | 63 | 28.2 | 26.6–29.7 |

**Additional file 32 Pooled prevalence of severity signs among only inpatients of vivax malaria (15 studies)**

| Complication | Total vivax | Total patients with severity sign | Pooled prevalence, % | 95% CI, % |
| --- | --- | --- | --- | --- |
| Death | 1367 | 63 | 28.2 | 26.6–29.7 |
| Cerebral malaria | 1367 | 122 | 10.1 | 5.6–14.7 |
| Multiple convulsions | 1372 | 18 | 13.7 | 11.5–15.8 |
| Renal dysfunction | 1367 | 97 | 5.9 | 0–14.1 |
| Respiratory dysfunction | 1367 | 46 | 1 | 0.1–1.8 |
| Hepatic dysfunction | 1367 | 262 | 19.5 | 12.6–26.3 |
| Abnormal bleeding/DIC | 1372 | 51 | 10.1 | 5.9–14.3 |
| Haemoglobinuria | 1367 | 65 | 1.9 | 0–7 |
| Hypoglycaemia | 1367 | 16 | 1.7 | 0–3.6 |
| Metabolic acidosis | 1367 | 100 | 7.8 | 0–23.3 |
| Circulatory collapse/Shock | 1367 | 32 | 5.7 | 0–13.3 |
| Severe anaemia | 1367 | 177 | 17.3 | 9.1–25.4 |
| Severe thrombocytopaenia | 1367 | 134 | 13.9 | 0–29.1 |
