# Supplementary material for: Severe vivax malaria: a systematic review and meta-analysis of clinical studies since 1900
Source: Malar J. 2014 Dec 8;13:481. doi: 10.1186/1475-2875-13-481 (PMC4364574; doi:10.1186/1475-2875-13-481)
Supplement: Supplementary file 16 — Additional file 16: Prevalence of severe thrombocytopenia among both outpatients and inpatients of vivax malaria. (DOCX 40 KB) [file 12936_2014_3678_MOESM16_ESM.docx]

**Additional file 16. Prevalence of severe thrombocytopenia among both outpatients and inpatients of vivax malaria**

| **Author (Reference)** | **Year** | **Country** | **Study design** | **Total vivax** | **Severe thrombocytopenia** | **Prevalence** | **95% CI** |
| --- | --- | --- | --- | --- | --- | --- | --- |
| Lynk[[27](#_ENREF_27)] | 1989 | Canada | RHBS | 24 | 2 | 8.33 | 1.03–27.0 |
| Lee [[32](#_ENREF_32)] | 1997 | Thailand | PHBS | 24 | 3 | 12.5 | 2.7–32.4 |
| Oh [[35](#_ENREF_35)] | 2001 | S. Korea | RHBS | 101 | 5 | 4.9 | 1.6–11.2 |
| Song [[38](#_ENREF_38)] | 2003 | S. Korea | RHBS | 44 | 9 | 20.4 | 9.8–35.3 |
| Sharma [[45](#_ENREF_45)] | 2009 | India | RHBS | 221 | 13 | 5.9 | 3.2–9.8 |
| Poespoprodjo[[43](#_ENREF_43)] | 2009 | Indonesia | PHBS | 102 | 23 | 22.5 | 14.9–31.9 |
| Singh [[59](#_ENREF_59)] | 2011 | India | RHBS | 108 | 9 | 8.3 | 3.9–15.2 |
| Mitja[[54](#_ENREF_54)] | 2011 | PNG | PHBS | 1213 | 3 | 0.2 | 0.05–0.7 |
| Shaikh [[66](#_ENREF_66)] | 2012 | Pakistan | RHBS | 192 | 19 | 9.9 | 6.1–15.0 |
| Mehmood[[68](#_ENREF_68)] | 2012 | Pakistan | RHBS | 97 | 44 | 45.4 | 35.2–55.8 |
| Naha [[15](#_ENREF_15)] | 2012 | India | RHBS | 213 | 68 | 32.0 | 26.0–39.0 |
| Sharma [[69](#_ENREF_69)] | 2012 | India | RHBS | 105 | 62 | 59.0 | 49.0–68.5 |
| Tanwar[[64](#_ENREF_64)] | 2012 | India | PHBS | 380 | 144 | 37.9 | 33.0–43.0 |
| Garg [[60](#_ENREF_60)] | 2012 | India | PHBS | 78 | 2 | 2.6 | 0.3–9.0 |
| Barber [[72](#_ENREF_72)] | 2013 | Malaysia | PHBS | 43 | 19 | 44.2 | 29.1–60.1 |
| Singh [[73](#_ENREF_73)] | 2013 | India | PHBS | 61 | 18 | 29.51 | 18.52–42.57 |
| Leal-Santos [[80](#_ENREF_80)] | 2013 | Brazil | PHBS | 186 | 31 | 16.66 | 11.61–22.81 |
| Bhatacharjee[[82](#_ENREF_82)] | 2013 | India | RHBS | 168 | 12 | 7.14 | 3.74–12.14 |
| Sarkar [[84](#_ENREF_84)] | 2013 | India | PHBS | 900 | 24 | 2.67 | 1.72–3.94 |
| Aatif[[86](#_ENREF_86)] | 2013 | Pakistan | PHBS | 107 | 96 | 89.72 | 82.34–94.75 |
| Kwak[[88](#_ENREF_88)] | 2013 | South Korea | RHBS | 352 | 148 | 42.04 | 36.83–47.39 |
| Pooled |  |  |  | 44478 | 754 | 7.5 | 4.2–10.8 |
