# Supplementary material for: Severe vivax malaria: a systematic review and meta-analysis of clinical studies since 1900
Source: Malar J. 2014 Dec 8;13:481. doi: 10.1186/1475-2875-13-481 (PMC4364574; doi:10.1186/1475-2875-13-481)
Supplement: Supplementary file 17 — Additional file 17: Prevalence of death among both outpatients and inpatients of vivax malaria. (DOCX 52 KB) [file 12936_2014_3678_MOESM17_ESM.docx]

**Additional file 17. Prevalence of death among both outpatients and inpatients of vivax malaria**

| **Author (Reference)** | **Year** | **Country** | **Study design** | **Total vivax** | **Death** | **Prevalence** | **95% CI** |
| --- | --- | --- | --- | --- | --- | --- | --- |
| Bahr [[18](#_ENREF_18)] | 1928 | USA | PHBS | 100 | 3 | 3.0 | 0.6–8.5 |
| Fitz-Hugh [[20](#_ENREF_20)] | 1944 | India | RHBS | 1375 | 1 | 0.1 | 0.002–0.4 |
| Whorton[[24](#_ENREF_24)] | 1947 | USA | PHBS | 195 | 1 | 0.5 | 0.01–2.8 |
| Singh [[28](#_ENREF_28)] | 1992 | India | PHBS | 25 | 1 | 4.0 | 0.1–20.3 |
| Wattanagoon[[29](#_ENREF_29)] | 1994 | Thailand | RHBS | 460 | 1 | 0.22 | 0.005–1.2 |
| Svenson[[30](#_ENREF_30)] | 1995 | Canada | RHBS | 246 | 1 | 0.4 | 0.01–2.2 |
| Vicas[[39](#_ENREF_39)] | 2005 | USA | RHBS | 30 | 1 | 3.3 | 0.1–17.2 |
| Barcus[[12](#_ENREF_12)] | 2007 | Indonesia | RHBS | 1135 | 9 | 0.8 | 0.4–1.5 |
| Beg [[41](#_ENREF_41)] | 2008 | Pakistan | RHBS | 270 | 4 | 1.5 | 0.4–3.7 |
| Tjitra[[13](#_ENREF_13)] | 2008 | Indonesia | PHBS | 2937 | 46 | 1.6 | 1.1–2.1 |
| Poespoprodjo[[43](#_ENREF_43)] | 2009 | Indonesia | PHBS | 668 | 6 | 0.9 | 0.3–1.9 |
| Kochar[[47](#_ENREF_47)] | 2009 | India | PHBS | 456 | 2 | 0.4 | 0.05–1.6 |
| Sharma [[45](#_ENREF_45)] | 2009 | India | RHBS | 221 | 3 | 1.4 | 0.3–3.9 |
| Kochar[[48](#_ENREF_48)] | 2010 | India | PHBS | 103 | 4 | 3.9 | 1.1–9.6 |
| Andrade [[49](#_ENREF_49)] | 2010 | Brazil | PHBS | 129 | 6 | 4.6 | 1.7–9.8 |
| Singh [[59](#_ENREF_59)] | 2011 | India | RHBS | 108 | 1 | 0.9 | 0.02–5.0 |
| Shaikh [[66](#_ENREF_66)] | 2012 | Pakistan | RHBS | 192 | 1 | 0.5 | 0.01–2.9 |
| Sharma [[69](#_ENREF_69)] | 2012 | India | RHBS | 105 | 10 | 9.5 | 4.7–16.8 |
| Limaye[[16](#_ENREF_16)] | 2012 | India | RHBS | 338 | 6 | 1.8 | 0.6–3.8 |
| Nurleila[[71](#_ENREF_71)] | 2012 | Indonesia | RHBS | 1837 | 18 | 1.0 | 0.6–1.5 |
| Garg [[60](#_ENREF_60)] | 2012 | India | PHBS | 78 | 1 | 1.3 | 0.03–6.9 |
| Singh [[73](#_ENREF_73)] | 2013 | India | PHBS | 61 | 5 | 8.2 | 2.7–18.1 |
| Zaki[[74](#_ENREF_74)] | 2013 | India | RHBS | 133 | 1 | 0.75 | 0.02–4.12 |
| Douglas [[75](#_ENREF_75)] | 2013 | Indonesia | RHBS | 19858 | 112 | 0.6 | 0.5–0.7 |
| Sarkar [[84](#_ENREF_84)] | 2013 | India | PHBS | 900 | 40 | 4.44 | 3.19–6.0 |
| Aatif[[86](#_ENREF_86)] | 2013 | Pakistan | PHBS | 107 | 1 | 0.93 | 0.02–5.1 |
| Rizvi [[87](#_ENREF_87)] | 2013 | India | RHBS | 172 | 3 | 1.74 | 0.36–5.01 |
| Jain [[89](#_ENREF_89)] | 2013 | India | PHBS | 198 | 2 | 1.01 | 0.122–3.6 |
| Pooled |  |  |  | 45044 | 290 | 0.2 | 0.1–0.3 |
