# Supplementary material for: Severe vivax malaria: a systematic review and meta-analysis of clinical studies since 1900
Source: Malar J. 2014 Dec 8;13:481. doi: 10.1186/1475-2875-13-481 (PMC4364574; doi:10.1186/1475-2875-13-481)
Supplement: Supplementary file 18 — Additional file 18: Pooled prevalence of severity signs among both inpatients and outpatients of vivax malaria (62 studies). (DOCX 24 KB) [file 12936_2014_3678_MOESM18_ESM.docx]

**Additional file 18 Pooled prevalence of severity signs among both inpatients and outpatients of vivax malaria (62 studies)**

| Complication | Total vivax | Total patients with severity sign | Pooled prevalence, % | 95% CI, % |
| --- | --- | --- | --- | --- |
| Death | 45044 | 290 | 0.2 | 0.1–0.3 |
| Cerebral malaria | 44478 | 410 | 0.6 | 0.3–0.8 |
| Multiple convulsions | 45014 | 70 | 0.1 | 0–0.3 |
| Renal dysfunction | 44478 | 147 | 0.5 | 0.1–0.8 |
| Respiratory dysfunction | 45044 | 98 | 0.1 | 0–0.3 |
| Hepatic dysfunction | 45169 | 465 | 2.5 | 1.7–3.4 |
| Abnormal bleeding/DIC | 44974 | 155 | 0.5 | 0.1–0.8 |
| Haemoglobinuria | 45044 | 28 | 1.4 | 0.4–2.4 |
| Hypoglycaemia | 45014 | 28 | 2 | 0.8–3.2 |
| Metabolic acidosis | 44478 | 38 | 0.4 | 0.1–0.7 |
| Circulatory collapse/Shock | 45044 | 35 | 3.3 | 1.1–5.4 |
| Severe anaemia | 45044 | 2099 | 2.8 | 1.8–3.9 |
| Severe thrombocytopaenia | 44478 | 754 | 7.5 | 4.2–10.8 |
