# Supplementary material for: Severe vivax malaria: a systematic review and meta-analysis of clinical studies since 1900
Source: Malar J. 2014 Dec 8;13:481. doi: 10.1186/1475-2875-13-481 (PMC4364574; doi:10.1186/1475-2875-13-481)
Supplement: Supplementary file 19 — Additional file 19: Prevalence of cerebral malaria among only inpatients of vivax malaria. (DOCX 34 KB) [file 12936_2014_3678_MOESM19_ESM.docx]

**Additional file 19. Prevalence of cerebral malaria among only inpatients of vivax malaria**

| Author (Reference) | Year | Country | Study design | Total vivax | Cerebral malaria | Prevalence | 95% CI |
| --- | --- | --- | --- | --- | --- | --- | --- |
| George [[50](#_ENREF_50)] | 2010 | India | RHBS | 30 | 2 | 6.7 | 0.8–22.1 |
| Manning [[51](#_ENREF_51)] | 2011 | PNG | PHBS | 27 | 7 | 25.9 | 11.1–46.3 |
| Yadav [[65](#_ENREF_65)] | 2012 | India | RHBS | 131 | 22 | 16.8 | 10.8–24.3 |
| Mahgoub[[61](#_ENREF_61)] | 2012 | Sudan | PHBS | 18 | 2 | 11.1 | 1.4–34.7 |
| Nadkar[[63](#_ENREF_63)] | 2012 | India | PHBS | 488 | 40 | 8.2 | 5.9–11 |
| Lanca[[67](#_ENREF_67)] | 2012 | Brazil | RHBS | 24 | 5 | 20.8 | 7.1–42.1 |
| Lon [[76](#_ENREF_76)] | 2013 | Cambodia | RHBS | 33 | 16 | 48.5 | 30.8–66.4 |
| Abdallah [[77](#_ENREF_77)] | 2013 | Sudan | PHBS | 26 | 3 | 11.54 | 2.45–30.15 |
| Sharma [[78](#_ENREF_78)] | 2013 | India | RHBS | 54 | 10 | 18.52 | 9.2–31.43 |
| Gehlawat[[79](#_ENREF_79)] | 2013 | India | PHBS | 18 | 9 | 50 | 26.02–73.98 |
| Zubairi[[85](#_ENREF_85)] | 2013 | Pakistan | RHBS | 296 | 6 | 2.03 | 0.75–4.36 |
| Pooled |  |  |  | 1367 | 122 | 10.1 | 5.6–14.7 |
