# Supplementary material for: Severe vivax malaria: a systematic review and meta-analysis of clinical studies since 1900
Source: Malar J. 2014 Dec 8;13:481. doi: 10.1186/1475-2875-13-481 (PMC4364574; doi:10.1186/1475-2875-13-481)
Supplement: Supplementary file 20 — Additional file 20: Prevalence of repeated generalized seizures among only inpatients of vivax malaria. (DOCX 29 KB) [file 12936_2014_3678_MOESM20_ESM.docx]

**Additional file 20. Prevalence of repeated generalized seizures among only inpatients of vivax malaria**

| Author (Reference) | Year | Country | Study design | Total vivax | Repeated generalized seizures | Prevalence | 95% CI |
| --- | --- | --- | --- | --- | --- | --- | --- |
| Mahgoub[[61](#_ENREF_61)] | 2012 | Sudan | PHBS | 18 | 3 | 16.7 | 3.6–41.4 |
| Lon [[76](#_ENREF_76)] | 2013 | Cambodia | RHBS | 33 | 2 | 6.06 | 0.74–20.23 |
| Abdallah [[77](#_ENREF_77)] | 2013 | Sudan | PHBS | 26 | 1 | 3.85 | 0.1–19.637 |
| Sharma [[78](#_ENREF_78)] | 2013 | India | RHBS | 54 | 4 | 7.41 | 2.05–17.89 |
| Gehlawat[[79](#_ENREF_79)] | 2013 | India | PHBS | 18 | 8 | 44.44 | 21.53–69.24 |
| Pooled |  |  |  | 1372 | 18 | 13.7 | 11.5–15.8 |
