# Supplementary material for: Severe vivax malaria: a systematic review and meta-analysis of clinical studies since 1900
Source: Malar J. 2014 Dec 8;13:481. doi: 10.1186/1475-2875-13-481 (PMC4364574; doi:10.1186/1475-2875-13-481)
Supplement: Supplementary file 21 — Additional file 21: Prevalence of renal dysfunction among only inpatients of vivax malaria. (DOCX 29 KB) [file 12936_2014_3678_MOESM21_ESM.docx]

**Additional file 21. Prevalence of renal dysfunction among only inpatients of vivax malaria**

| **Author (Reference)** | **Year** | **Country** | **Study design** | **Total vivax** | **Renal dysfunction** | **Prevalence** | **95% CI** |
| --- | --- | --- | --- | --- | --- | --- | --- |
| George [[50](#_ENREF_50)] | 2010 | India | RHBS | 30 | 1 | 3.3 | 0.1–17.2 |
| Manning [[51](#_ENREF_51)] | 2011 | PNG | PHBS | 27 | 1 | 3.7 | 0.1–19.0 |
| Yadav [[65](#_ENREF_65)] | 2012 | India | RHBS | 131 | 8 | 6.1 | 2.7–11.7 |
| Lanca[[67](#_ENREF_67)] | 2012 | Brazil | RHBS | 24 | 2 | 8.3 | 1.0–27.0 |
| Nandwani[[70](#_ENREF_70)] | 2012 | India | RHBS | 110 | 75 | 68.2 | 58.6–76.7 |
| Zubairi[[85](#_ENREF_85)] | 2013 | Pakistan | RHBS | 296 | 10 | 3.38 | 1.63–6.12 |
| Pooled |  |  |  | 1367 | 97 | 5.9 | 0–14.1 |
