# Supplementary material for: Severe vivax malaria: a systematic review and meta-analysis of clinical studies since 1900
Source: Malar J. 2014 Dec 8;13:481. doi: 10.1186/1475-2875-13-481 (PMC4364574; doi:10.1186/1475-2875-13-481)
Supplement: Supplementary file 22 — Additional file 22: Prevalence of respiratory dysfunction among only inpatients of vivax malaria. (DOCX 32 KB) [file 12936_2014_3678_MOESM22_ESM.docx]

**Additional file 22. Prevalence of respiratory dysfunction among only inpatients of vivax malaria**

| **Author (Reference)** | **Year** | **Country** | **Study design** | **Total vivax** | **Respiratory dysfunction** | **Prevalence** | **95% CI** |
| --- | --- | --- | --- | --- | --- | --- | --- |
| George [[50](#_ENREF_50)] | 2010 | India | RHBS | 30 | 1 | 3.3 | 0.1–17.2 |
| Nayak[[52](#_ENREF_52)] | 2011 | India | PHBS | 80 | 4 | 5.0 | 1.4–12.3 |
| Nadkar[[63](#_ENREF_63)] | 2012 | India | PHBS | 488 | 8 | 1.6 | 0.7–3.2 |
| Yadav [[65](#_ENREF_65)] | 2012 | India | RHBS | 131 | 3 | 2.3 | 0.5–6.5 |
| Lanca[[67](#_ENREF_67)] | 2012 | Brazil | RHBS | 24 | 3 | 6.7 | 2.7–32.4 |
| Lon [[76](#_ENREF_76)] | 2013 | Cambodia | RHBS | 33 | 1 | 3.03 | 0.08–15.76 |
| Sharma [[78](#_ENREF_78)] | 2013 | India | RHBS | 54 | 3 | 5.55 | 1.16–15.39 |
| Zubairi[[85](#_ENREF_85)] | 2013 | Pakistan | RHBS | 296 | 23 | 7.77 | 4.99–11.43 |
| Pooled |  |  |  | 1367 | 46 | 1.0 | 0.1–1.8 |
