# Supplementary material for: Severe vivax malaria: a systematic review and meta-analysis of clinical studies since 1900
Source: Malar J. 2014 Dec 8;13:481. doi: 10.1186/1475-2875-13-481 (PMC4364574; doi:10.1186/1475-2875-13-481)
Supplement: Supplementary file 23 — Additional file 23: Prevalence of hepatic dysfunction among only inpatients of vivax malaria. (DOCX 36 KB) [file 12936_2014_3678_MOESM23_ESM.docx]

**Additional file 23. Prevalence of hepatic dysfunction among only inpatients of vivax malaria**

| **Author (Reference)** | **Year** | **Country** | **Study design** | **Total vivax** | **Hepatic dysfunction** | **Prevalence** | **95% CI** |
| --- | --- | --- | --- | --- | --- | --- | --- |
| Rodriguez-Morales [[46](#_ENREF_46)] | 2009 | Venezuela | RHBS | 17 | 6 | 35.3 | 14.2–61.7 |
| George [[50](#_ENREF_50)] | 2010 | India | RHBS | 30 | 13 | 43.3 | 25.5–62.6 |
| Nayak[[52](#_ENREF_52)] | 2011 | India | PHBS | 80 | 23 | 28.75 | 19.18–39.95 |
| Mahgoub[[61](#_ENREF_61)] | 2012 | Sudan | PHBS | 18 | 5 | 27.8 | 9.7–53.5 |
| Nadkar[[63](#_ENREF_63)] | 2012 | India | PHBS | 488 | 95 | 19.5 | 16.0–23.3 |
| Lanca[[67](#_ENREF_67)] | 2012 | Brazil | RHBS | 24 | 6 | 8.9 | 9.8–46.7 |
| Nandwani[[70](#_ENREF_70)] | 2012 | India | RHBS | 110 | 60 | 54.5 | 44.8–64.1 |
| Lon [[76](#_ENREF_76)] | 2013 | Cambodia | RHBS | 33 | 4 | 12.12 | 3.40–28.2 |
| Abdallah [[77](#_ENREF_77)] | 2013 | Sudan | PHBS | 26 | 3 | 11.54 | 2.45–30.15 |
| Sharma [[78](#_ENREF_78)] | 2013 | India | RHBS | 54 | 14 | 25.92 | 14.96–39.65 |
| Gehlawat[[79](#_ENREF_79)] | 2013 | India | PHBS | 18 | 5 | 27.78 | 9.69–53.5 |
| Zubairi[[85](#_ENREF_85)] | 2013 | Pakistan | RHBS | 296 | 28 | 9.46 | 6.38–13.38 |
| Pooled |  |  |  | 1367 | 262 | 19.5 | 12.6–26.3 |
