# Supplementary material for: Severe vivax malaria: a systematic review and meta-analysis of clinical studies since 1900
Source: Malar J. 2014 Dec 8;13:481. doi: 10.1186/1475-2875-13-481 (PMC4364574; doi:10.1186/1475-2875-13-481)
Supplement: Supplementary file 24 — Additional file 24: Prevalence of abnormal bleeding/DIC among only inpatients of vivax malaria. (DOCX 31 KB) [file 12936_2014_3678_MOESM24_ESM.docx]

**Additional file 24. Prevalence of abnormal bleeding/DIC among only inpatients of vivax malaria**

| **Author (Reference)** | **Year** | **Country** | **Study design** | **Total vivax** | **Abnormal bleeding/DIC** | **Prevalence** | **95% CI** |
| --- | --- | --- | --- | --- | --- | --- | --- |
| Rodriguez-Morales [[46](#_ENREF_46)] | 2009 | Venezuela | RHBS | 17 | 4 | 23.5 | 6.8–49.9 |
| Yadav [[65](#_ENREF_65)] | 2012 | India | RHBS | 131 | 12 | 9.1 | 4.8–15.4 |
| Mahgoub[[61](#_ENREF_61)] | 2012 | Sudan | PHBS | 18 | 2 | 11.1 | 1.4–34.7 |
| Lon [[76](#_ENREF_76)] | 2013 | Cambodia | RHBS | 33 | 5 | 15.15 | 5.11–31.9 |
| Abdallah [[77](#_ENREF_77)] | 2013 | Sudan | PHBS | 26 | 2 | 7.69 | 0.94–25.13 |
| Sharma [[78](#_ENREF_78)] | 2013 | India | RHBS | 54 | 10 | 18.52 | 9.25–31.43 |
| Zubairi[[85](#_ENREF_85)] | 2013 | Pakistan | RHBS | 296 | 16 | 5.4 | 3.12–8.63 |
| Pooled |  |  |  | 1372 | 51 | 10.1 | 5.9–14.3 |
