# Supplementary material for: Severe vivax malaria: a systematic review and meta-analysis of clinical studies since 1900
Source: Malar J. 2014 Dec 8;13:481. doi: 10.1186/1475-2875-13-481 (PMC4364574; doi:10.1186/1475-2875-13-481)
Supplement: Supplementary file 25 — Additional file 25: Prevalence of haemoglobinuria among only inpatients of vivax malaria. (DOCX 26 KB) [file 12936_2014_3678_MOESM25_ESM.docx]

**Additional file 25. Prevalence of haemoglobinuria among only inpatients of vivax malaria**

| **Author (Reference)** | **Year** | **Country** | **Study design** | **Total vivax** | **Hemoglobinuria** | **Prevalence** | **95% CI** |
| --- | --- | --- | --- | --- | --- | --- | --- |
| Lanca[[67](#_ENREF_67)] | 2012 | Brazil | RHBS | 24 | 2 | 8.3 | 1.0–27.0 |
| Sharma [[78](#_ENREF_78)] | 2013 | India | RHBS | 54 | 1 | 1.85 | 0.047–9.89 |
| Zubairi[[85](#_ENREF_85)] | 2013 | Pakistan | RHBS | 296 | 62 | 20.95 | 16.45–26.03 |
| Pooled |  |  |  | 1367 | 65 | 1.9 | 0–7.0 |
