# Supplementary material for: Severe vivax malaria: a systematic review and meta-analysis of clinical studies since 1900
Source: Malar J. 2014 Dec 8;13:481. doi: 10.1186/1475-2875-13-481 (PMC4364574; doi:10.1186/1475-2875-13-481)
Supplement: Supplementary file 26 — Additional file 26: Prevalence of hypoglycaemia among only inpatients of vivax malaria. (DOCX 29 KB) [file 12936_2014_3678_MOESM26_ESM.docx]

**Additional file 26. Prevalence of hypoglycaemia among only inpatients of vivax malaria**

| **Author (Reference)** | **Year** | **Country** | **Study design** | **Total vivax** | **Hypoglycemia** | **Prevalence** | **95% CI** |
| --- | --- | --- | --- | --- | --- | --- | --- |
| Mahgoub[[61](#_ENREF_61)] | 2012 | Sudan | PHBS | 18 | 5 | 27.8 | 9.7–53.5 |
| Yadav [[65](#_ENREF_65)] | 2012 | India | RHBS | 131 | 1 | 0.7 | 0.02–4.2 |
| Lanca[[67](#_ENREF_67)] | 2012 | Brazil | RHBS | 24 | 3 | 12.5 | 2.7–32.4 |
| Nandwani[[70](#_ENREF_70)] | 2012 | India | RHBS | 110 | 2 | 1.8 | 0.2–6.4 |
| Abdallah [[77](#_ENREF_77)] | 2013 | Sudan | PHBS | 26 | 2 | 5.23 | 0.94–25.13 |
| Zubairi[[85](#_ENREF_85)] | 2013 | Pakistan | RHBS | 296 | 3 | 1.01 | 0.21–2.93 |
| Pooled |  |  |  | 1367 | 16 | 1.7 | 0–3.6 |
