# Supplementary material for: Severe vivax malaria: a systematic review and meta-analysis of clinical studies since 1900
Source: Malar J. 2014 Dec 8;13:481. doi: 10.1186/1475-2875-13-481 (PMC4364574; doi:10.1186/1475-2875-13-481)
Supplement: Supplementary file 27 — Additional file 27: Prevalence of metabolic acidosis among only inpatients of vivax malaria. (DOCX 28 KB) [file 12936_2014_3678_MOESM27_ESM.docx]

**Additional file 27. Prevalence of metabolic acidosis among only inpatients of vivax malaria**

| **Author (Reference)** | **Year** | **Country** | **Study design** | **Total vivax** | **Metabolic acidosis** | **Prevalence** | **95% CI** |
| --- | --- | --- | --- | --- | --- | --- | --- |
| Manning [[51](#_ENREF_51)] | 2011 | PNG | PHBS | 27 | 3 | 11.1 | 2.3–29.2 |
| Lanca[[67](#_ENREF_67)] | 2012 | Brazil | RHBS | 24 | 10 | 41.7 | 22.1–63.4 |
| Nandwani[[70](#_ENREF_70)] | 2012 | India | RHBS | 110 | 70 | 63.6 | 53.9–72.6 |
| Zubairi[[85](#_ENREF_85)] | 2013 | Pakistan | RHBS | 296 | 17 | 5.74 | 3.38–9.04 |
| Pooled |  |  |  | 1367 | 100 | 7.8 | 0–23.3 |
