# Supplementary material for: Severe vivax malaria: a systematic review and meta-analysis of clinical studies since 1900
Source: Malar J. 2014 Dec 8;13:481. doi: 10.1186/1475-2875-13-481 (PMC4364574; doi:10.1186/1475-2875-13-481)
Supplement: Supplementary file 28 — Additional file 28: Prevalence of shock among only inpatients of vivax malaria. (DOCX 29 KB) [file 12936_2014_3678_MOESM28_ESM.docx]

**Additional file 28. Prevalence of shock among only inpatients of vivax malaria**

| **Author (Reference)** | **Year** | **Country** | **Study design** | **Total vivax** | **Shock** | **Prevalence** | **95% CI** |
| --- | --- | --- | --- | --- | --- | --- | --- |
| Lanca[[67](#_ENREF_67)] | 2012 | Brazil | RHBS | 24 | 13 | 54.2 | 32.9–74.4 |
| Lon [[76](#_ENREF_76)] | 2013 | Cambodia | RHBS | 33 | 7 | 21.21 | 8.98–38.91 |
| Abdallah [[77](#_ENREF_77)] | 2013 | Sudan | PHBS | 26 | 4 | 15.38 | 4.36–34.87 |
| Gehlawat[[79](#_ENREF_79)] | 2013 | India | PHBS | 18 | 3 | 16.67 | 3.58–41.42 |
| Zubairi[[85](#_ENREF_85)] | 2013 | Pakistan | RHBS | 296 | 5 | 1.69 | 0.55–3.9 |
| Pooled |  |  |  | 1367 | 32 | 5.7 | 0–13.3 |
