# Supplementary material for: Severe vivax malaria: a systematic review and meta-analysis of clinical studies since 1900
Source: Malar J. 2014 Dec 8;13:481. doi: 10.1186/1475-2875-13-481 (PMC4364574; doi:10.1186/1475-2875-13-481)
Supplement: Supplementary file 29 — Additional file 29: Prevalence of severe anaemia among only inpatients of vivax malaria. (DOCX 34 KB) [file 12936_2014_3678_MOESM29_ESM.docx]

**Additional file 29. Prevalence of severe anaemia among only inpatients of vivax malaria**

| **Author (Reference)** | **Year** | **Country** | **Study design** | **Total vivax** | **Severe anemia** | **Prevalence** | **95% CI** |
| --- | --- | --- | --- | --- | --- | --- | --- |
| George [[50](#_ENREF_50)] | 2010 | India | RHBS | 30 | 3 | 10.0 | 2.1–26.5 |
| Manning [[51](#_ENREF_51)] | 2011 | PNG | PHBS | 27 | 4 | 14.9 | 4.2–33.7 |
| Nayak[[52](#_ENREF_52)] | 2011 | India | PHBS | 80 | 26 | 32.5 | 22.4–43.9 |
| Mahgoub[[61](#_ENREF_61)] | 2012 | Sudan | PHBS | 18 | 6 | 33.3 | 13.3–59.0 |
| Yadav [[65](#_ENREF_65)] | 2012 | India | RHBS | 131 | 45 | 34.3 | 26.3–43.1 |
| Lanca[[67](#_ENREF_67)] | 2012 | Brazil | RHBS | 24 | 4 | 16.7 | 4.7–37.4 |
| Nandwani[[70](#_ENREF_70)] | 2012 | India | RHBS | 110 | 65 | 59.1 | 49.3–68.4 |
| Lon [[76](#_ENREF_76)] | 2013 | Cambodia | RHBS | 33 | 11 | 33.33 | 17.96–51.83 |
| Sharma [[78](#_ENREF_78)] | 2013 | India | RHBS | 54 | 8 | 14.81 | 6.62–27.12 |
| Gehlawat[[79](#_ENREF_79)] | 2013 | India | PHBS | 18 | 5 | 27.78 | 9.69–53.48 |
| Pooled |  |  |  | 1367 | 177 | 17.3 | 9.1–25.4 |
