# Supplementary material for: Severe vivax malaria: a systematic review and meta-analysis of clinical studies since 1900
Source: Malar J. 2014 Dec 8;13:481. doi: 10.1186/1475-2875-13-481 (PMC4364574; doi:10.1186/1475-2875-13-481)
Supplement: Supplementary file 30 — Additional file 30: Prevalence of severe thrombocytopenia among only inpatients of vivax malaria. (DOCX 29 KB) [file 12936_2014_3678_MOESM30_ESM.docx]

**Additional file 30. Prevalence of severe thrombocytopaenia among only inpatients of vivax malaria**

| **Author (Reference)** | **Year** | **Country** | **Study design** | **Total vivax** | **Severe thrombocytopenia** | **Prevalence** | **95% CI** |
| --- | --- | --- | --- | --- | --- | --- | --- |
| George [[50](#_ENREF_50)] | 2010 | India | RHBS | 30 | 28 | 93.3 | 77.9–99.2 |
| Yadav [[65](#_ENREF_65)] | 2012 | India | RHBS | 131 | 17 | 13.0 | 7.7–20.0 |
| Lanca[[67](#_ENREF_67)] | 2012 | Brazil | RHBS | 24 | 7 | 29.2 | 12.6–51.1 |
| Mahgoub[[61](#_ENREF_61)] | 2012 | Sudan | PHBS | 18 | 4 | 22.2 | 6.4–47.6 |
| Sharma [[78](#_ENREF_78)] | 2013 | India | RHBS | 54 | 20 | 37.04 | 24.29–51.26 |
| Zubairi[[85](#_ENREF_85)] | 2013 | Pakistan | RHBS | 296 | 58 | 19.59 | 15.23–24.58 |
| Pooled |  |  |  | 1367 | 134 | 13.9 | 0–29.1 |
