# Supplementary material for: Severe vivax malaria: a systematic review and meta-analysis of clinical studies since 1900
Source: Malar J. 2014 Dec 8;13:481. doi: 10.1186/1475-2875-13-481 (PMC4364574; doi:10.1186/1475-2875-13-481)
Supplement: Supplementary file 31 — Additional file 31: Prevalence of death among only inpatients of vivax malaria. (DOCX 34 KB) [file 12936_2014_3678_MOESM31_ESM.docx]

**Additional file 31. Prevalence of death among only inpatients of vivax malaria**

| **Author (Reference)** | **Year** | **Country** | **Study design** | **Total vivax** | **Death** | **Prevalence** | **95% CI** |
| --- | --- | --- | --- | --- | --- | --- | --- |
| Giglioli[[19](#_ENREF_19)] | 1930 | British Guiana | RHBS | 15 | 3 | 20.0 | 4.3–48.1 |
| Rodriguez-Morales  [[46](#_ENREF_46)] | 2009 | Venezuela | RHBS | 17 | 1 | 5.9 | 0.1–28.7 |
| George [[50](#_ENREF_50)] | 2010 | India | RHBS | 30 | 2 | 6.7 | 0.8–22.1 |
| Manning [[51](#_ENREF_51)] | 2011 | PNG | PHBS | 27 | 1 | 3.7 | 0.1–19.0 |
| Nadkar[[63](#_ENREF_63)] | 2012 | India | PHBS | 488 | 44 | 9.0 | 6.6–11.9 |
| Yadav [[65](#_ENREF_65)] | 2012 | India | RHBS | 131 | 4 | 3.0 | 0.8–7.6 |
| Lanca[[67](#_ENREF_67)] | 2012 | Brazil | RHBS | 24 | 2 | 8.3 | 1.0–27.0 |
| Lon [[76](#_ENREF_76)] | 2013 | Cambodia | RHBS | 33 | 4 | 12.12 | 3.4–28.2 |
| Gehlawat[[79](#_ENREF_79)] | 2013 | India | PHBS | 18 | 2 | 11.11 | 1.37–34.7 |
| Pooled |  |  |  | 1367 | 63 | 28.2 | 26.6–29.7 |
