# Supplementary material for: Severe vivax malaria: a systematic review and meta-analysis of clinical studies since 1900
Source: Malar J. 2014 Dec 8;13:481. doi: 10.1186/1475-2875-13-481 (PMC4364574; doi:10.1186/1475-2875-13-481)
Supplement: Supplementary file 32 — Additional file 32: Pooled prevalence of severity signs among only inpatients of vivax malaria (15 studies). (DOCX 24 KB) [file 12936_2014_3678_MOESM32_ESM.docx]

**Additional file 32 Pooled prevalence of severity signs among only inpatients of vivax malaria (15 studies)**

| Complication | Total vivax | Total patients with severity sign | Pooled prevalence, % | 95% CI, % |
| --- | --- | --- | --- | --- |
| Death | 1367 | 63 | 28.2 | 26.6–29.7 |
| Cerebral malaria | 1367 | 122 | 10.1 | 5.6–14.7 |
| Multiple convulsions | 1372 | 18 | 13.7 | 11.5–15.8 |
| Renal dysfunction | 1367 | 97 | 5.9 | 0–14.1 |
| Respiratory dysfunction | 1367 | 46 | 1 | 0.1–1.8 |
| Hepatic dysfunction | 1367 | 262 | 19.5 | 12.6–26.3 |
| Abnormal bleeding/DIC | 1372 | 51 | 10.1 | 5.9–14.3 |
| Haemoglobinuria | 1367 | 65 | 1.9 | 0–7 |
| Hypoglycaemia | 1367 | 16 | 1.7 | 0–3.6 |
| Metabolic acidosis | 1367 | 100 | 7.8 | 0–23.3 |
| Circulatory collapse/Shock | 1367 | 32 | 5.7 | 0–13.3 |
| Severe anaemia | 1367 | 177 | 17.3 | 9.1–25.4 |
| Severe thrombocytopaenia | 1367 | 134 | 13.9 | 0–29.1 |
